# Artificial intelligence applied in neoantigen identification facilitates personalized cancer immunotherapy

**DOI:** 10.3389/fonc.2022.1054231

**Published:** 2023-01-09

**Authors:** Yu Cai, Rui Chen, Shenghan Gao, Wenqing Li, Yuru Liu, Guodong Su, Mingming Song, Mengju Jiang, Chao Jiang, Xi Zhang

**Affiliations:** ^1^ School of Medicine, Northwest University, Xi’an, Shaanxi, China; ^2^ Department of Neurology, The Second Affiliated Hospital of Xi’an Medical University, Xi’an, Shaanxi, China

**Keywords:** neoantigen prediction, cancer neoantigen, cancer immunotherapy, artificial intelligence, next generation sequencing

## Abstract

The field of cancer neoantigen investigation has developed swiftly in the past decade. Predicting novel and true neoantigens derived from large multi-omics data became difficult but critical challenges. The rise of Artificial Intelligence (AI) or Machine Learning (ML) in biomedicine application has brought benefits to strengthen the current computational pipeline for neoantigen prediction. ML algorithms offer powerful tools to recognize the multidimensional nature of the omics data and therefore extract the key neoantigen features enabling a successful discovery of new neoantigens. The present review aims to outline the significant technology progress of machine learning approaches, especially the newly deep learning tools and pipelines, that were recently applied in neoantigen prediction. In this review article, we summarize the current state-of-the-art tools developed to predict neoantigens. The standard workflow includes calling genetic variants in paired tumor and blood samples, and rating the binding affinity between mutated peptide, MHC (I and II) and T cell receptor (TCR), followed by characterizing the immunogenicity of tumor epitopes. More specifically, we highlight the outstanding feature extraction tools and multi-layer neural network architectures in typical ML models. It is noted that more integrated neoantigen-predicting pipelines are constructed with hybrid or combined ML algorithms instead of conventional machine learning models. In addition, the trends and challenges in further optimizing and integrating the existing pipelines are discussed.

## 1 Introduction

In recent years, neoantigen-based immunotherapy has received widespread attention. Neoantigens are abnormal proteins produced by cancer cells through non-synonymous mutations, which specifically bind to MHC molecules and present to the surface of cancer cells or antigen presenting cells. The presented neoantigens are recognized by T cells and the activated T cells would attack and eliminate cancer cells. Tumor neoantigens have become an ideal target for immunotherapy because they will not cause central immune tolerance or autoimmune diseases (1). Rapid and accurate neoantigen identification plays a crucial role in effective individualized cancer immunotherapy. The traditional method for identifying neoantigens, using cDNA library screening, is labor intensive, costly, and cannot effectively identify all tumor antigens. The advent of next-generation sequencing (NGS) technology and bioinformatics make it possible to rapidly identify tumor-specific mutations on a large scale and screen neoantigens. Most current neoantigen prediction methods focus on peptide processing and presentation predicting peptides, which can be presented to the surface of tumor cells. However, additional *in vitro* experiments are needed to verify whether these predicted peptides are immunogenic or not. Finally, only a few verified neoantigens can elicit a T-cell response. Thus, there is a need for mature, accurate, and systematic pipelines that can predict meaningful immunogenic neoantigens.

AI is the capacity of a machine to study and identify characteristics from input data, and can use learned information to make decisions on new data (2). AI mainly consists of conventional ML and deep learning. The main principle of AI is to input large structured or unstructured data, which is then trained by an AI system and produce outputs the prediction of new targets. Due to the characteristics of large amounts of input data and deep training layers, AI has shown the advantage of high accuracy in several biomedical fields. For example, Lee et al. (3) developed an artificially intelligent tactile ferroelectric skin (ATFES), which can perceive and learn various tactile information at the same time. This study showed a credible and multiplex batch of essential synaptic functions were confirmed based on ATFES, including the excellent cycling stability during 10 000 continuous electrical input pulses and low variability (3.18%). The accuracy of this test is higher than 99% even after considering 10% noise. AI is also showing promising trends in the application of genome analysis, such as evaluating fragmentation patterns of cell-free DNA across the genome using ML (4), predicting splicing from the primary sequence using deep learning algorithms (5), identifying sequence context features predictive of transcription factor binding using deep neural networks (6), and variant calling using a Bayesian model (7). AI can also improve approaches for protein studies, such as prediction of multi-level peptide-protein interaction using deep learning algorithms (8), and the establishment of immunoinformatic tool using ML to predict tumor T-cell antigens (9).

This review offers first introduces the overall workflow for neoantigen prediction and the application of machine learning tools in each steps of this workflow. We restricted our scope to the feature extraction algorithms by which the large-scale input datasets are processed, recognized and transited into neoantigen features. Then we detailed the integrated pipelines that either implemented by ML tools or an end-to-end deep learning model. The former ones usually are developed to focus on extracting peptide-MHC (pMHC) binding affinity as key features while the latter ones tend to include immunogenicity characterization as additional layer of feature to train the model. The final section addresses concern the possible problems and predicts the development trends of ML-based application in this field.

## 2 Data source for model training and testing

Currently, most *in silico* algorithms in neoantigen prediction are based on ML and the performance is highly dependent on the training data. As shown in **Table S1**, the typical data source used for construction of ML-based somatic mutation callers are summarized. Validated Mass Spectrum (MS)-derived MHC-binding sequences, such as from Immune Epitope Database (IEDB database), are the most common data used to train models that predict novel neoantigen with peptide-MHC binding feature. The reason is that the single measured binding data is merely indicative of binding event (10). Moreover, single peptide detected from MS are not necessarily neoantigens, as some detected peptides may also be expressed in normal cells, which may be subject to central tolerance. Known TCR-peptide pairs sequences or TCR-peptide pairs sequences with certain HLA alleles or sequences were employed to establish ML-based approaches to predict the interaction of TCR and peptide or pMHC. Another example of common data in neoantigen predictor training is experimentally tested immunogenic epitopes and nonimmunogenic epitopes with wild type and mutant specificity. It is believed ML is powerful to solve the increasingly complex data required to identify new neoantigen. These datasets will be divided proportionally into training data and test data or be split into a K number of folds (K-fold cross validation) where each fold is used as a training or testing set at some point (11).

From the **Table S1**, the available training data is limited (mostly from IEDB), which may lead to potential sampling biases and may lead to poor predicting performance. There is also a notable imbalance issue as the positive case is extremely less than negative data, which leads to the biases on classifier trained on these data. Some solutions are proposed to alleviate these situations. For example, data simulation and simulated datasets may address the imbalance issue (12). The synthetic minority over-sampling technique was developed (SMOTE) (13) and employed (14) to reduce the imbalance impact by over-sampling approach. Overall, the data validity of IEDB is decent as it has been opened and maintained to public for many years and thus has been validated and confirmed by numerous independent studies. Currently, there is relatively few neoantigens data derived from limited tumor types and biased ethnicity groups. Therefore, additional database or resource built at a pan-cancer and multi-ethnic (mainly from western country) setting is greatly expected and will benefit this field. In addition, it would be beneficial to collect data or construct some datasets collecting epitopes from species (15) covers MHC molecules from non-human primates. The biases of established methods due to the biases in the data would be improve by using single data (including single cancer type, ethnic, and specie) and increasing the amount of corresponding data in the future. On top of improving the diversity and validity of the data source, the model training and testing would also be improved *via* novel, advanced and integrated machine learning algorithms. For instance, long short-term memory neural network architecture that has been used for handling problem of peptide length (16). In addition, *in vitro* validation experiments need to be improved to avoid data omission issues.

## 3 Feature extraction algorithms in neoantigen prediction

**Figure 1:**
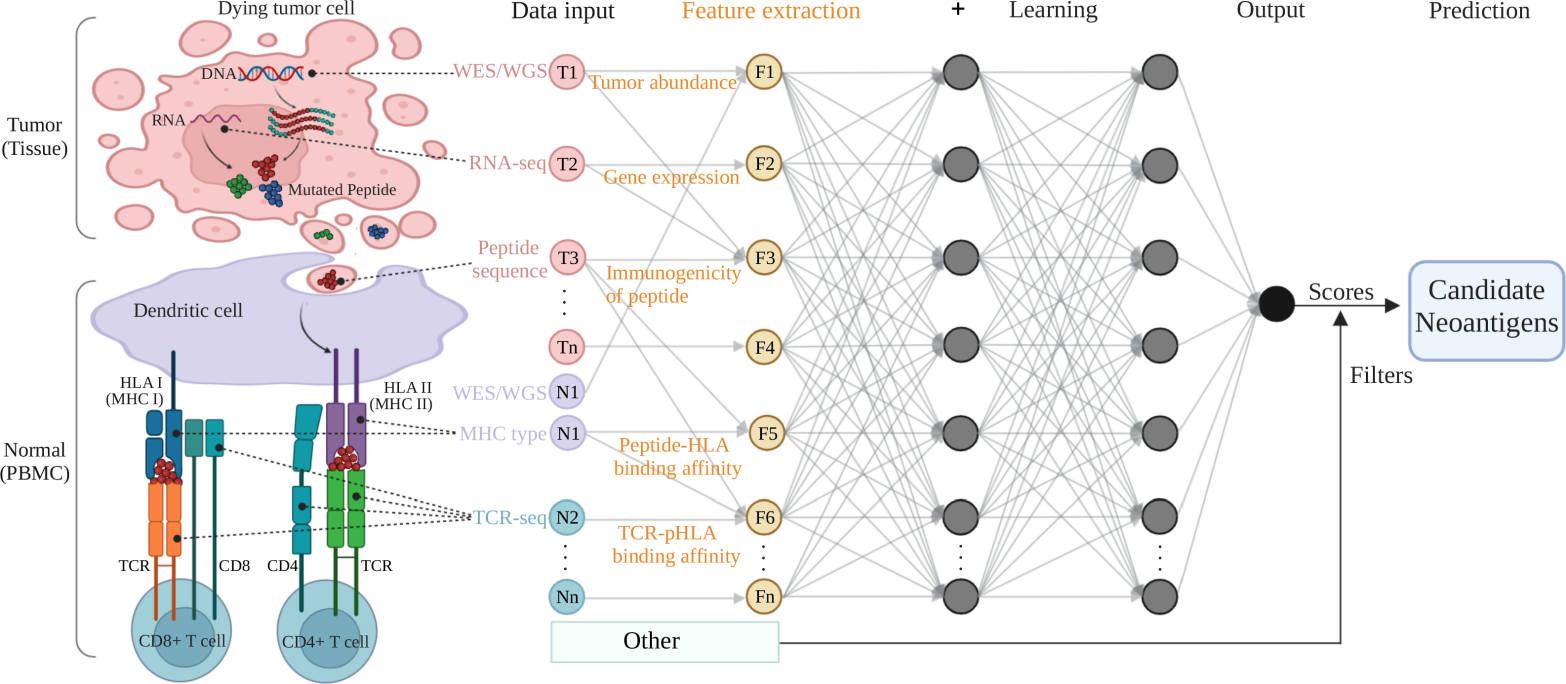
A typical multi-layer neural network architecture showing the feature extraction and prediction procedures for neoantigen predicting. Tumor (tissue) and normal (PBMC) samples are processed by NGS-based genomic profiling (WES/WGS etc.) and undergo bioinformatic analysis to produce input data for machine learning training. The key features (tumor abundance etc.) extracted from the input data are fed to a typical neural network (deep learning) model and filters to output a predicting score (or a predicted value) for the class (positive or negative for neoantigen) the input data belongs to. The colored circles represent the input data collected from tumor components (pink, T1 to Tn) and normal cells or immune receptors (purple or sapphire, N1 to Nn), as well as the feature variables (yellow, F1 to Fn) extracted from the omics data input. The gray arrows that connect the circles shows how all the neurons are interconnected and stacked together to constitute a layer, and the multiple layers piled next to each other to construct the neural network model. Figure was created with BioRender.com.

The goal of neoantigen prediction is to identify immunogenic epitopes that binding to MHC molecules to elicit T cells immune responses. Previous neoantigen prediction methods mainly considered the binding affinity of peptide-MHC, which require a large number of *in vitro* or *in vivo* validation experiments to test the immunogenicity of the predicted candidate neoantigens and only few validated peptides were immunogenic. The most of current neoantigen prediction methods not only consider peptide-MHC binding affinity but also the immunogenicity by incorporating the immunogenicity related features into the filters or AI models in the workflow of neoantigen recognition (**Figure 1**).

The typical feature extraction algorithms are mainly used in the five steps of neoantigen prediction including somatic mutation calling, MHC typing, assessment of peptide-HLA binding affinity, TCR-pMHC binding prediction and prediction of the immunogenicity of candidate neoantigens. And the algorithms used in the most of these steps are commonly based on AI model to obtain the high prediction accuracy. The current general neoantigen prediction workflow involves multiple steps. Matched normal-tumor WGS/WES data were analyzed to identify tumor somatic mutations. Corresponding tumor RNA-seq data were used to select candidate neoantigens according to gene expression levels. Not all mutations cause new epitopes to be recognized by the immune system due to HLA limitations (17). Therefore, HLA typing needed to be considered when predicting potential neoantigens. A patient’s HLA alleles can be determined by DNA and RNA sequencing data or PCR techniques (18). Next, mutated peptide sequences and patient’s HLA alleles were analyzed using informatics tools or an AI model to predict HLA-peptide binding affinity. This was done to further rank candidate neoantigens as those that may present on a tumor cell surface by MHC class I or antigen-presenting cells by MHC class II. However, the predicted candidate neoantigens may not elicit immune responses from T cells. Therefore, TCR-pMHC binding and the immunogenicity related features need to be considered for identifying true immunogenic neoantigens.

### 3.1 Variant calling

Somatic mutations that generate neoantigens mainly include single nucleotide variants (SNVs), small insertions and deletions (indels) (19), gene fusions (20), intron retentions (21), exon-exon junctions (22), splice variants (23), noncoding regions (24), and human endogenous retroviruses (25). In recent years, researchers proved that peptides deriving from unannotated open reading frames (nuORFs) identified by Ribo-Seq can be presented to the tumor cell surface by MHC I as extra sources of tumor neoantigens (26).

#### 3.1.1 SNV

Currently, neoantigens derived from SNVs are still the most common source studied and validated by many independent studies. However, only a small percentage of predicted neoantigens are identified as immunogenic peptides due to the significant similarity to the normal one. Compared with SNV-derived neoantigens, neoantigens derived from non-SNV variants are more immunogenic due to non-SNV variants’ high potential to change protein sequences (27). However, limited tools are available to identify non-SNV variants and the low accuracy of these tools preclude non-SNV variant derived neoantigen prediction.

The basic workflow of somatic variant calling includes several steps: 1) bam files undergo mapping, alignment and cleaning; 2) variant calling in reads from WES/WGS or targeted sequencing of the paired tumor and normal samples; 3) somatic variant filtering including removal of germline variants; 4) copy number and structural variant calling may require additional sources and tools. Notably, it is a challenging as somatic mutations always present at low frequency which made them difficult to detect (28). Usually, matched tumor and normal samples are used for somatic variant calling. It is essential to use high purity samples to perform DNA sequencing. High levels of normal DNA “contamination” in tumor sample and tumor DNA “contamination” in normal para-cancerous tissue sample sometimes decreases the sensitivity of somatic variant calling (29). Compared to para-cancerous tissue, the PBMCs were frequently used by researchers as “blank” or “baseline” sample due to the low tumor DNA “contamination”. Therefore, it is best to use matched tumor and normal adjacent samples for somatic variants calling to minimize the false-positive. However, it is not always feasible for researchers to obtain paired human samples. Identification of somatic variants from tumor-only DNA sequencing would increase the challenging of germline variants filtering which is also dependent on the individual’s ancestry (30). It should to be noted that to correct for genetic ancestry related germline false positives in absence of matched normal. Halperin et al. (31) developed a Bayesian tumor-only somatic variant caller (LumosVar) that employs the allelic frequency difference between somatic and germline mutations in tumor samples to call somatic variant, which seems greatly reduce false positives and avoid bias introduced by variants from genetic ancestry. However, the sensitivity of LumosVar heavily relies on a number of factors including the purity of tumor samples and enough sequencing depth. At present, such methods are still relatively rare and require further development.

The tumor heterogeneity makes it more difficult to develop a solid caller. The incorporation of ML into the mutation identification has been proved to improve variant calling performance. Numbers of machine learning-based variant callers (**Table S2**) have been developed to identify genetic variations. Each tool has a certain scope of application. Among these tools, DeepVariant (32), a machine learning-based variant caller, developed by the Google Brain team, aims to identify genetic variation in sequencing data by constructing an image classification model using deep neural networks. It is the first to apply neural networks to the detection of biological sequence variants. In short, it encodes the aligned read and reference data as an image for each candidate mutation site. Then, a trained CNN image recognition model based on a tensorflow deep learning framework was used to compute the genotype likelihoods for each site to find genetic variants from high-throughput sequencing data to perform genotyping. The PrecisionFDA Truth Challenge had reported that the DeepVariant was the most accurate variant caller compared with other existing variant callers, with 99.96% (SNVs) and 99.40% (short indels) of F-score values. And Supernat et al. compared the DeepVariant tool to the commonly used GATK 4.0 and SpeedSeq (33), finding that DeepVariant outperforms GATK, a golden standard pipeline. However, the open source DeepVariant takes a very long time, almost twice as long as GATK, which largely limits its applications. Accordingly, Huang et al. (34) shows a DeepVariant-on-Spark optimizes resource distribution, and reduces the time needed to process the DeepVariant pipeline.

Cerebro, developed by Wood et al. (12), is a machine learning-based somatic mutation discovery approach. Cerebro employs a random forest that evaluates a large set of decision trees to produce a confidence score for every candidate mutation. Normal peripheral blood DNA sample was used to train the model. Somatic mutations that mutant allele fractions arrange from 1.5 to 100% and a real-world representative source were used as a training data. Compared with the existing methods (sensitivity: 90 to 99%, positive predictive value: 34 to 92%) in recognizing experimentally validated variants, Cerebro (sensitivity: 97%, positive predictive value: 98%) has better performance. And this method is totally automated without the need for expert to supervise sequence data. However, Cerebro is unsustainable for widespread NGS analyses.

NeuSomatic is a tool that uses a deep convolutional neural network for somatic variant calling (35). Nine convolutional layers are constructed in the network architecture. NeuSomatic uses a new input matrice that include alignment information to train the model, which allows feature extraction directly from the raw sequencing data. On the final layer, two softmax classifiers and one regressor were used to predict the mutation. The accuracy of the method on real data show that NeuSomatic performs better than NeuSomatic-S due to the fact that the former integrates the outputs and intermediate results of other detection methods into the input, while NeuSomatic-S only uses the raw sequencing data as the input. In terms of run time, the NeuSomatic is 3.5 times longer than the NeuSomatic-S. In addition, the tool is written in PyTorch, supports GPUs, and takes only 156 core-CPU hours to train the data (30X) compared to the 1000 CPU core-hours required by DeepVariant. NeuSomatic performs significantly better than the state-of-the-art variant calling algorithms, specifically, for identifying samples in low tumor purities and allelic frequencies.

Although there are many somatic variant callers, no one is perfect, and accuracy may be low when using a single tool to identify a variant. Thus, using multiple variants callers simultaneously to determine potential tumor somatic mutations could potentially increase the accuracy of the calling (36). After identifying the variants, the commonly used annotators, such as Variant Effect Predictor (VEP) (37), ANNOVAR (38) and snpEff (39) are used to annotate them to help us determine the effect of nucleotide changes on the sequence of the coding protein. ANNOVAR is designed for annotation of gene-based coding change, especially variants in classic databases such as dbSNP/1000 Genome Project etc. VEP is an open tool that used for annotation of variants in both coding and non-coding genomic regions. In contrast to ANNOVAR which only provides gene-level annotations, VEP offers transcript-level annotations as well as mutations in species in addition to human. The unique feature of SnpEff and the derivative tool SnpSift is they use optimized algorithm to efficiently tackle variant annotation problems including annotation process standardization, protein change calculation and loss of function evaluation (40). Nevertheless, once the mutated amino acid sequences are successfully annotated, the next step would be to evaluate their binding affinity with MHC class I/II (41).

#### 3.1.2 Alternative variation sources

In addition to SNVs and small indels as traditional variant source for novel neoantigen identification, other variation types, such as gene fusions (20), alternative splicing variants and so on, were confirmed to trigger immune cell responses. Notably, the accuracy of somatic variant callers for non-SNVs mutations was not low. For instance, the fusion genes lead to chimeric proteins that were highly immunogenic due to their unique structure. STAR-Fusion (42), recommended by National Cancer Institute, is a software for fusion identification based on STAR alignment results. Fusion-Bloom (43), employs the recent advance in *de novo* transcriptome assembly and assembly-based structural mutation identification methods to identify fusions. Compared with the other fusion identification tools, Fusion-Bloom showed an improved sensitivity and specificity in identify real fusion variants. Haas et al. (44) assessed the performance of 23 gene fusion callers and demonstrated STAR-SEQR, STAR-Fusion, and Arriba have the best performance in identifying novel gene fusions from cancer transcriptomes. Due to the lack of exposure in prior studies, neoantigen candidates derived from fusion and other uncommon variation source are perhaps more immunogenic and make better targets for immunotherapy.

### 3.2 HLA typing

The important function of MHC class I molecules is to participate in the process of antigen presentation to CD8+ cytolytic T cells. MHC class II molecules mainly present processed exogenous antigen fragments to CD4+ T cells during the initial stage of immune response. MHC class I molecules have a peptide-binding groove where the two ends are closed, limiting the size of their ligands to about 8-11aminoacids (45). The antigen binding sites mainly target the backbone of antigen peptides with relatively conserved amino acid sequences, which facilitate the binding of the variable amino acid side chains in the free state on the antigen peptide and the TCR. Contrary to MHC class I, class II molecules have open binding groove that allows them to bind longer peptides, typically 12-20 amino acid residues in length (46). The complex open binding groove brings about difficultly in evaluating binding affinity between antigens and MHC class II.

Predicting HLA typing is essential for identifying neoantigens. Different MHC haplotypes have different binding affinity with peptides, so it is crucial to accurately genotype the patient’s HLA alleles before peptides-MHC binding affinity prediction. Polymerase chain reaction-sequencing based typing (PCR-SBT) is the gold standard for HLA genotyping (47). However, there are a large number of alleles in HLA, and the polymorphisms of alleles are outside the analysis region, or the alleles are heterozygous, which may lead to ambiguous genotyping results. With the advancements in NGS technology, it is possible to perform HLA allele typing *in silico*, which provides an economical and efficient approach. Studies have reported that certain HLA class I typing tools can achieve up to 99% accuracy compared with the gold standard method. The existing available computational tools are shown in **Table S3**, Optitype, Polysolver, seq2HLA, and PHLAT are widely employed algorithms to call MHC I alleles or II alleles using DNA or RNA sequencing data. Polysolver is currently one of the recognized standard tools for working with low-coverage WES data. Many studies indicate that Optitype, with high specificity and selectivity, is the most accurate tool to genotype HLA class I alleles using WES data or RNA-seq data (48). PHLAT performs best when predicting both Class I and II allele genotypes, and seq2HLA is the best choice to deal with RNA-seq data (49).

### 3.3 Peptide-MHC binding prediction

#### 3.3.1 Peptide-MHC I binding prediction

Peptide-MHC binding is an important feature source to screen neoantigens that can be used for clinical immunotherapy. The tools (**Table S4**) used for the prediction of peptide-MHC binding affinity have been developed. Different strategies have been used to construct the tools. Initially, tools were mainly based on linear regression and stabilized matrix approaches including Pickpocket. A linear contribution to the peptide-MHC binding affinity by each of amino acid was considerate in Linear regression. Currently, the state-of-the-art prediction methods mainly rely on machine learning algorithms. The machine learning can identify the nonlinear connection of the peptide sequence and MHC molecules *via* the network layers. In addition, machine learning-based prediction tools use a large amount of data from binding affinity data or/and mass spectrometry (MS) peptidome data to train models and have higher accuracy than linear regression-based tools.

NetMHC (50) is an allele-specific epitope prediction tool employing an artificial neural network to predict peptide-MHC binding affinity. This method needs to divide the training data according to alleles. Therefore, it is difficult to accurately predict the alleles with insufficient training data. In addition, viral peptides and most frequent HLA-alleles (such as HLA-A∗02:01) are used as training data for constructing this tool, which introduce the bias of selection of viral-like peptides and tumor antigens that preferred presented by frequent HLA molecules. Recently, the version of this tool has been upgraded to NetMHC 4.0 (51), which now includes additional alleles. It should be noted that most HLA molecules are preferred to bind 9 mers of peptide. NetMHCpan 4.0 (15), the newest version of the pan-specific tool based on artificial neural networks, is trained on a combination of more than 180,000 quantitative binding affinity data and mass spectrometry peptidome data, showing the highest accuracy in predicting peptide-MHC binding affinity (52). O’Donnell et al. presents an allele-specific neural networks-based MHC I binding predictor, MHCflurry (53). The software employs a new architecture and peptide encoding strategy. MHCflurry exceeds NetMHC 4.0 and NetMHCpan 3.0 when trained on binding affinity data. The newest MHCflurry 2.0 (54) includes two experimental predictors, an “antigen processing” predictor and a “presentation” predictor. An “antigen processing” predictor tries to model MHC allele-independent factors such as proteasomal cleavage. A “presentation” predictor combines processing predictions with binding affinity predictions to generate a presentation score. However, they only used MS datasets of MHC class I to train and evaluate their methods. The accuracy scores may be inflated by modeling assay biases through the AP predictor.

Hu et al. (55) proposed an interpretable pan-specific peptide-MHC I binding affinity prediction model, ACME. The model combines a deep convolutional neural network with an attention module. First, ACME performs initial feature extraction of the encoded peptide and MHC pseudo-sequences through a convolutional layer, and then maps the extracted features to the convolutional module and the attention module. After that, the outputs of these two modules are combined for the final prediction. Extensive tests showed that ACME (SRCC: 0.569) outperformed other methods, including NetMHCpan 2.8 (SRCC: 0.512), NetMHCpan 3.0 (SRCC: 0.522) and NetMHCpan 4.0 (SRCC: 0.521). However, the contribution of the attention module is limited. Besides, they only employed limited experimental data to evaluate the different methods. IConMHC (56) is a pan-allele method with a CNN model for the prediction of peptide-MHC binding affinity. Unlike other methods, iConMHC studies physical and chemical interaction properties (such as contact potentials and distances) of pairwise amino acids from the peptide and MHC molecule. Before putting the input data into the iConMHC model, each pair of peptide and MHC were processed into a 3D matrix that acts as the input data. The dimension of the 3D matrix is 48 × 9 × 19, where the width and height of each slice represents the length of peptide (9 amino acid long) and MHC (48 amino acid long), respectively, and each of the 19 slices (depth) represents amino acid interaction of peptide and MHC. In the iConMHC model, two convolutional layers were used to train data. The first layer includes 32 filters with a 3 × 1 max pooling layer behind it, and the second layer includes 64 filters with a 2 × 1 max pooling layer behind it. ReLu activation function is used in two layers. The second layer connected to a layer that connects the output neuron. The iConMHC model captures features from the interaction information making peptide-MHC binding prediction. The benchmarking result showed that iConMHC performs better than most of the pan-allele models but has a similar SRCC score to netMHC3.4. Anthem, developed by Mei et al. (57), has a novel two-layer prediction structure. The first layer uses five scoring functions commonly used in peptide and HLA-I binding prediction to synthetically code the amino acids sequence of each peptide and extract features of amino acid sequences. In the second layer, a machine learning model, aggregating one-dependence estimators (AODE) trained by features obtained from the first layer, is used for binding prediction. Anthem has been proven to outperform NetMHCpan 4.1 and MixMHCpred 2.0.2 using independent datasets. And Anthem has the best AUC value on 12 HLA I allotypes for all peptide lengths. However, compared with other tools, the AUC values of Anthem were the lowest when predicting certain HLA-I types including HLA-A∗02:17, HLA-A∗02:50, and HLA-A∗24:06 for 9-mer peptides.

Due to the multiple polymorphism of HLA alleles and the lack of experimental data of binding affinity for many HLA alleles, it is to date still very difficult to develop a model for each allele (58). Therefore, at present, the binding affinity threshold of existing predictors is specified to all HLA-alleles. For example, the threshold for strong binder of all peptides-HLA is specified as 0.5 (%rank) and for weak binder is 2 (%rank) as described in NetMHCpan and other similar tools from NetMHC family. In contrast to common HLA alleles, which have been studied tested extensively in training prediction algorithms, the HLA alleles with low allele frequency is limited by the resource of training data, which may result potential bias in prediction algorithms. Therefore, it’s necessary to specify an optimal threshold for each type of HLA-allele to improve the prediction accuracy of peptide-HLA binding affinity. In addition, it is notable that peptide-MHC binding prediction algorithms seem to have low prediction ability for peptides containing certain amino acids. For instance, it is common that protein cysteine can be oxidized and disulfide bonds can be formed between cysteine residues under oxidizing settings, which potentially interfere the binding of cysteine-containing peptides to HLA molecules (59). This is the reason why certain amino acids sometimes are under-represented in training data, which eventually introduce bias for peptides containing cysteine. Studies have showed that bioinformatic (60) or chemical method (61) can be used to partially correct for the loss of cysteine-containing peptides. Therefore, it is very necessary to adopt appropriate strategy to improve the accuracy of prediction algorithms for peptides containing under-represented amino acids like cysteine.

Faced with a large number of such prediction tools, researchers need to choose the optimal tools for their own research. However, prediction performance differs depending on the MHC type and peptide length. Therefore, it is necessary to evaluate the existing peptide-MHC binding prediction algorithms to help people to choose the best tool or tools for their studies. Bonsack et al. (18) evaluated the prediction performance of 13 peptide-MHC binding predictors. They found that artificial neural networks-based pan-specific methods showed the highest prediction accuracy in general. Similarly, Paul et al. evaluated the performance of 17 available Peptide-MHC binding predictors (62). The results showed that neural network-based NetMHCPan-4.0 and MHCFlurry have the best performance. Mei et al. (63) recently performed a comprehensive review and performance evaluation of 15 tools for peptide-HLA I binding prediction. The results showed that MixMHCpred 2.0.1 has the best performance to predict peptide-HLA binding, while NetMHCpan 4.0 outperforms the other machine learning-based methods, and NetMHCcons 1.1 surpass consensus-based tools. Overall, methods that based on ML models including NetMHCpan 4.0, MHCflurry, and so on performing the better performance on binding affinity prediction compared with other methods. As machine learning-based methods continue to be developed, more methods should be evaluated to provide researchers with better options.

#### 3.3.2 Peptide-MHC II binding prediction

It has been reported that neoantigens presented by MHC II have a crucial function in anti-tumor response (10). However, compared with peptide-MHC I binding predictors, algorithms for predicting peptide-MHC II are fewer and the prediction accuracy of currently peptide-MHC II binding predictors is still low. Unlike MHC I, the peptide length recognized by MHC II is highly variable. The reason is that the binding groove of MHC II is open at two ends, allowing the bonded peptides up to 30 amino acids (10). This structure characteristic of MHC II and little available training data are main causes in making accurate peptide-MHC II binding predictions difficult. In recent years, with the production of related data, some methods have been developed to predict peptide-MHC II binding, such as NetMHCII (64), TEPITOPEpan (65), NetMHCIIpan (66), and RANKPEP (67). NetMHCII and NetMHCIIpan, ANN-based methods, have been demonstrated to have higher performance in predicting peptide-MHC II binding than other tools (68, 69). The newest version of NetMHCIIpan is NetMHCIIpan-4.0, which utilizes ANN algorithm to predict peptide-MHC II binding. NetMHCIIpan-4.0 is trained on over 500,000 binding affinity data and eluted ligand mass spectrometry data (70). Compared to the older versions, NetMHCIIpan-4.0 has significantly improved predictive performance, underlining the necessity of expanding training data.

Degoot et al. (58) presented a trans-allelic prediction model to predict the interaction of peptide-MHC II. This model was trained using a dataset including quantitative binding data that was employed to develop NetMHCIIpan-3.0 (71). This method provided a reasonable physical explanation for the interaction of peptide and MHC II, which is a notable advantage of this method compared with existing data-driven methods. This model has comparable performance to the intra-allele model on average for prediction of HLA-DP (0.930 vs. 0.928), HLA-DQ (0.830 vs. 0.857), and HLA-DR (0.780 vs. 0.771).

MHCnuggets as a long short-term memory (LSTM) deep neural network was recently built by Shao et al. (16) to predict binding affinity of peptide-MHC I or II. This neural network model was trained *via* binding affinity data for selective MHC II allele. Each neural network was composed of four layers including an input sequence layer, a LSTM layer with 64 hidden units, a fully connected layer with 64 hidden units, and an output layer of a single sigmoid unit. Compared to methods that combination of binding affinity data and mass spectrometry data, MHCnuggets showed a comparable prediction ability. For example, they employed a five-fold cross validation to evaluated the performance of MHCnuggets and NetMHCIIpan-3.2 as well as NetMHCII-2.3. The overall auROC for all 27 class II alleles was 0.849 for MHCnuggets, which was comparable to that of the NetMHCIIpan-3.2 (0.861) and NetMHCII-2.3 (0.861). However, this method is limited to analyze missense mutations.

DeepSeqPanII, a LSTM-CNN model with attention mechanism for prediction of peptide-HLA II binding, was developed by Liu et al. in 2022 year (72). The network structure is built by peptide sequence encoders, HLA α and β encoders, a context extractor, and a binding affinity predictor. The encoded HLA α and β chains, and a peptide were as input sample, which were put into LSTM block that outputted two kinds of hidden tensors. Then the two tensors were fed into the attention block. Three weighted outputs got from the attention blocks were combined with channel axis going into the convolutional network that outputs a 1D vector. Finally, it was put into a fully connected network to predict binding affinity of peptide-MHC II. Comparison with other existing peptide-HLAII binding predictors. DeepSeqPanII and NetMHCIIpan-3.1 have the best performance, however, the accuracy of DeepSeqPanII was still lower than NetMHCIIpan-3.1.

More predictors for calculating binding affinity of peptide-MHC II including MARIA and MixMHC2pred have been described by Moore et al. in review (73). At present, the accuracy of predictors for identification of peptide-MHC II binding affinity is significantly lower than that of peptide-MHC I. Hence, the most important things are to increase the training data of peptide-MHC II binding data and select appropriate ML model to learning the training data to improve the prediction accuracy of peptide-MHC II binding predictors.

### 3.4 TCR-pMHC binding prediction

A large number of candidates neoantigens can be obtained by predicting the binding affinity of peptide-MHC. However, whether all of these candidate neoantigens elicit an immune response from T cells requires additional *in vitro* experimental validations. With the ever-growing amount of available TCR-pMHC specificity data, methods used to predict the interaction between TCR and pMHC complexes have been developed in recent years. For instance, Springer et al. developed a TCR-peptide binding predictor, ERGO (pEptide tcR matchinG predictiOn), by combing large-scale TCR-peptide dictionaries with deep learning methods (74). ERGO studied a model for the whole set of peptides using a deep learning algorithm called long short-term memory. The CDR3 and peptides were encoded and used as input data to train a neural network. The output of 1 represented the TCR and peptide bind and 0 was otherwise. ERGO showed similar performance to state-of-the-art methods when using a set of standard tests. However, the MHC was not included in the model, which may influence the accuracy of the predictor.

NetTCR-2.0, a sequence-based method, used a CNN model to train paired TCRα and β sequence data for peptide-TCR binding prediction (75). The peptide, the CDR3α, and/or CDR3β sequences were used as inputs in the neural network and the BLOSUM50 matrix was used for encoding the amino acids. The encoded sequences were processed by a 1D convolutional layer and a max-pooling layer. Then, the extracted features were concatenated into a model constructed by dense layers. The output result was the binding probability of a peptide-TCR pair. The sigmoid function was used as the activation function in the network. Compared to other methods using an independent paired TCR dataset, NetTCR-2.0 perform the best, with a high specificity (identifying 79% of the positive TCR at a false-positive rate of 2%). NetTCR-2.0 is limited to analyzing HLA-A*02:01 and 9-mer peptides. However, it is an important step forward due to the consideration of not only TCRβ sequence data, but also TCRα sequence data.

Lu et al. (76) developed a transfer learning-based pMHC-TCR binding prediction network, pMTnet, to predict the TCR binding specificities of neoantigens presented by MHC I. The training and prediction of pMTnet is based on the sequence of mutually recognized antigens during T-cell-tumor cell binding, the MHC sequence and the TCR sequence. pMTnet applies transfer-learning to complete the training of the deep learning model. The AUC reached a high accuracy of 0.827 when using an independence testing dataset to evaluate the performance of pMTnet. pMTnet shows a significant improvement in prediction accuracy compared to other prediction models including TCRex, TCRGP, and netTCR. One potential problem in this study is that the existing biased representation of certain antigens and corresponding pairing TCRs in their training dataset.

Above all, the quality and quantity of training data are the key factors for AI-based model, which significantly influence the prediction accuracy of TCR-pMHC binding. The peptide characteristics and other information such as TCR sequencing coverage also helps to improve prediction accuracy. As more data are released, especially CDR3α sequence information, it will no longer be a challenge to accurately predict peptide and TCR interactions.

### 3.5 Prediction of the immunogenicity of candidate neoantigens

It is still unclear how epitopes elicit T cell immune responses. However, several studies found that epitope sequence-based features play important roles in T cell epitope immune response, elicited by epitope. These features include the molecular size of peptides (77), sequence similarity (78), hydrophobicity of amino acids at TCR contact residues (79), polarity of amino acids (80), entropy of peptides (81), and amino acid pairwise contact potentials (82), in addition to peptide-MHC binding affinity. AI or scoring systems are commonly used to treat the peptide sequencing-based features (we will introduce detailed information in section 4.2). Hence, a reasonable integration of features into the neoantigen identification pipeline would improve prediction accuracy.

## 4 Integrated machine learning-based pipelines for neoantigen prediction

**Figure 2:**
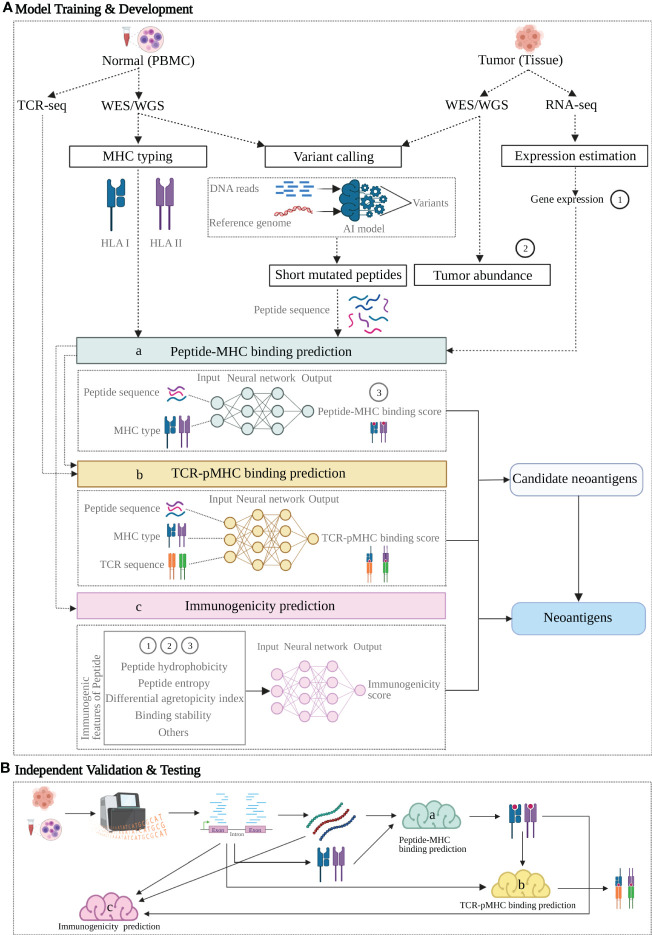
A proposed neoantigen-predicting workflow implemented with machine learning (ML) models targeting individual characteristics. A group of verified neoantigen data is split into two datasets for Training + Development and Testing respectively. **(A)** Upper dotted line box: Model Training and Development. 1) individual features of the training data with known class (positive or negative for neoantigen) are either produced from NGS profiling directly or indirectly as additional rounds of analysis may apply to generate the variables; 2) as indicated by dashed arrow lines, these feature variables act as input in three ML models (colored boxes) targeting three characteristics: peptide-MHC binding (model a, sapphire), TCR-pMHC binding (model b, yellow) and Immunogenicity (model c, pink); 3) as indicated by the arrow lines, each model learns from its own input data and generate a prediction or together produces an integrated prediction; 4) ML model compare its prediction against the true class of the training data and learn from this training, following by optimization aiming for a better prediction. **(B)** Lower dotted line box: Independent Validation and Testing. After the predictive models trained and developed, a candidate neoantigen will undergo NGS-based genomic profiling and generated input data, followed by processing in the three trained models (a-c, colored clouds) and eventually provide the predictions. Figure was created with BioRender.com.

A combination of machine learning algorithms helps to integrate end-to-end machine learning-based pipelines and enable the omics data to be processed and transformed into neoantigen prediction. In addition to using the assessment of peptide-MHC and peptide-TCR binding affinity to predict neoantigens, integrated pipelines with improved accuracy were also developed by researchers to recognize immunogenicity of the mutated peptides (**Figure 2**). Here, we focus on two strategies (i): Neoantigen prediction pipelines utilizing ML filters to extract peptide-HLA binding affinity as key feature (ii), Neoantigen prediction pipelines utilizing immunogenicity related features as input to train ML models for prediction of immunogenic neoantigens.

### 4.1 Prediction of neoantigens based on ML filter and other features

In fact, only about 2% of the neoantigens predicted by binding affinity were validated as immunogenic and able to elicit T cell immune responses in *in vitro* experiments (83). The lower prediction accuracy increases the amount of time spent and the total number of validation experiments. Moreover, most laboratories can only perform a limited number of validation experiments due to lack of funds. These challenges greatly limit the study and application of neoantigens in immunotherapy. In addition to binding affinity, other features, such as peptide cleavage information, binding affinity of peptides to TAP molecules and binding stability of peptides and MHC molecules are also involved in the process of neoantigen presentation, affecting the accuracy of the prediction. Thus, researchers added these potential factors into pipelines to improve the accuracy of neoantigen identification.

As shown in **Table 1**, we summarized recently developed neoantigen prediction pipelines based on peptide-MHC binding affinity and filters. Most pipelines are open source and do not require users to be skilled in bioinformatics, which is very convenient for researchers and clinicians. The pipelines in **Table 1** are complementary. For example, Epitoolkit (84) is a web-based platform for vaccine design, which offers immunoinformatics tools to predict neoantigens that could be used in vaccine development. Epitoolkit utilizes ANN-based NetMHC to calculate the peptide-MHC I binding scores. The pipeline incorporates proteasomal cleavage site predictions to filter mutated peptides. However, the quality of mutations from the aspect of the depth and coverage of sequencing data and the expression abundance of neoantigens were not consider, which would result in a false positive prediction. pVACseq (85), a pipeline based on step-by-step analysis of the filtering strategy for identifying and prioritizing neoantigens from DNA-seq and RNA-Seq data, uses ANN-based NetMHC v3.4 for prediction of MHC I restricted epitopes. And the pipeline employs information about sequence coverage, allele variation frequency, and gene expression to prioritize neoantigens. FRED 2 uses the parameters that Epitoolkit and pVACseq employs, including cleavage prediction, mutation coverage, mutated allele frequency and mutated gene expression plus transporter associated with antigen processing (TAP) prediction to identify neoantigens. Additionally, an advantage of FRED 2 (86) is that there are many available tools for users to choose at each step of neoantigen prediction including prediction of peptide-MHC binding by AI-based NetMHC, NetMHCpan, NetMHC II, and NetMHCpan II, which avoids modifying their data format and uses the most suitable tool to meet the overall design. Vaxrank (93) is a pipeline for predicting neoantigen from tumor variances, RNA data, and HLA type. ANN-based NetMHCpan was used to predict peptide-MHC affinity. Vaxrank employs a scoring system that combines *ExpressionScore* (gene expression) and *TotalBindingScore* (peptide-MHC affinity) to generate *RankingScore* for predicted mutant peptides ranking, which makes the results more likely to be the most promising neoantigens.

**Table 1:** Neoantigen prediction pipelines extracting peptide-HLA binding affinity as key feature.

Pipeline	Data input (Neoantigen source)	Filtering Strategy and Machine Learning Tool	Affinity type
EpiToolkit (84) (2015)	WGS/WES/RNA-seq (SNVs, indels)	(1) Peptide-MHC binding affinity (Tool: NetMHC [ANNs*]. Input: peptide sequence and HLA type) (2) Proteasomal cleavage (Tool: PCM and NetChop [neural network]. Input: peptide sequence)	MHC I
pVAC-Seq (85)(2016)	WES/RNA-seq(SNVs, indels)	(1) Peptide-MHC binding affinity (Tool: NetMHC v3.4 [ANNs]. Input: peptide sequence and HLA type) (2) Sequence coverage (3) Allele variation frequency (Tool: bam-readcount. Input: Tumor-DNA and RNA and normal-DNA) (4) Gene expression (Tool: Cufflinks. Input: RNA-seq reads)	MHC I
FRED 2 (86) (2016)	WES/RNA-seq (SNVs, indels)	(1) Proteasomal cleavage (Tool: ProteaSMM (C/S20), PCM, NetChop [neural network], and Ginodi. Input: peptide sequence) (2) Mutation coverage (3) Mutated allele frequency (4) Mutated gene expression (5) TAP transport efficiency (Tool: SVMTAP [SVM], SMMTAP, and Additive matrix method. Input: peptide sequence, HLA type, and peptide length) (6) Peptide-MHC binding affinity (Tool: NetMHC, NetMHCpan, NetMHC II, NetMHC II pan [ANNs]. Input: peptide sequence and HLA type)	MHC I and MHC II
CloudNeo (87) (2017)	Vcf file (for mutations) and bam file (for HLA typing) (SNVs)	Peptide-MHC binding affinity (Tool: NetMHCpan [ANNs]. Input: peptide sequence and HLA type)	MHC I
Tlminer (88) (2017)	RNA-seq (SNVs)	(1) Gene expression (Tool: Kallisto. Input: RNA-seq reads) (2) Peptide-MHC binding affinity (Tool: NetMHCpan [ANNs]. Input: peptide sequence and HLA type)	MHC I
TSNAD (89) (2017)	WES/RNA-seq (SNVs, indels)	Peptide-MHC binding affinity (Tool: NetMHCpan [ANNs]. Input: peptide sequence and HLA type)	MHC I
INTEGRATENeo (90) (2017)	WGS/RNA-seq (Gene fusions)	Peptide-MHC binding affinity (Tool: NetMHC4 [ANNs]. Input: peptide sequence and HLA type)	MHC I and MHC II
NeoantigenR (91) (2017)	DNA/RNA-seq (SNVs, indels, splicing variants)	Peptide-MHC binding affinity (Tool: NetMHC [ANNs]. Input: peptide sequence and HLA type)	MHC I
MuPeXI (92)	WES (SNVs, indels)	(1) Peptide-MHC binding affinity (Tool: NetMHCpan 3.0. Input: peptide sequence and HLA type) (2) Gene expression (3) The number of mismatches between the mutant and normal peptides. (4) Mutant allele frequency (5) Normal exact match penalty	MHC I
Vaxrank (93) (2018)	RNA-seq (SNVs, indels)	(1) Peptide-MHC binding affinity (Tool: NetMHCpan [ANNs]. Input: peptide sequence and HLA type) (2) Gene expression	MHC I
ScanNeo (94) (2019)	RNA-seq (indels)	(1) Peptide-MHC binding affinity (Tool: NetMHC [ANNs], NetMHCpan [ANNs]. Input: peptide sequence and HLA type) (2) Fold change between WT and MT alleles (3) Variant allele frequency (Tool: VEP. Input: identified indel)	MHC I
NeoPredPipe (95) (2019)	Somatic variant calls (SNVs, indels)	(1) Peptide-MHC binding affinity (Tool: NetMHCpan [ANNs]. Input: peptide sequence and HLA type) (2) Neoantigen recognition potentials (the amplitude of the ratio of the relative probabilities of binding for the wild-type and mutant epitopes to the MHC-class I molecules, and a measure of similarity to pathogenic peptides)	MHC I
Neoepiscope (96) (2019)	DNA-seq (SNVs, indels)	Peptide-MHC binding affinity (Tool: MHCflurry [neural network], NetMHCpan [ANNs]. Input: peptide sequence and HLA type)	MHC I and MHC II
pVACtools (97) (2020)	Somatic variant calls (SNVs, indels, gene fusions)	(1) Rank of binding affinity (Tool: MHCflurry [neural network], NetMHC [ANNs], NetMHCpan [ANNs], NetMHCIIpan [ANNs]. Input: peptide sequence and HLA type) (2) Rank of fold change between mutant and wild-type alleles (WT/MT binding affinity). (3) Rank of mutant allele expression (rank of gene expression * rank of mutant allele RNA variant allele fraction) (4) Rank of DNA variant allele fraction (Tool: VEP. Input: variants)	MHC I and MHC II
neoANT-HILL (98) (2020)	WGS/WES/RNA-seq (SNVs, indels)	(1) Gene expression (Tool: Kallisto. Input: RNA-seq reads) (2) Peptide-MHC binding affinity (Tool: MHCflurry [neural network], NetMHC [ANNs], NetMHCpan [ANNs], NetMHCIIpan [ANNs]. Input: peptide sequence and HLA type)	MHC I
NeoFuse (99) (2020)	RNA-seq (Gene fusions)	(1) Gene expression (Tool: STAR and featureCounts. Input: RNA-seq reads) (2) Peptide-MHC binding affinity (Tool: MHCflurry [neural network]. Input: peptide sequence and HLA type)	MHC I
ASNEO (100) (2020)	RNA-seq (Alternative splicing)	(1) Peptide-MHC binding affinity (Tool: NetMHCpan-4.0 [ANNs], Input: peptide sequence and HLA type) (2) The number of mismatches between the mutant peptide and normal peptide (3) The combined score of binding affinity, proteasomal C terminal cleavage and TAP transport efficiency of candidate neoantigen (Tool: NetCTLpan, Input: peptide sequence and HLA type) (4) Hydrophobicity score (machine-learning model, Input: peptide hydrophobicity information) (5) T cell recognition score	MHC I
VENUS (101) (2021)	WES and RNA sequencing data (SNVs and indels)	(1) Allele frequency of the mutations (2) Abundance of the transcripts carrying the mutation (3) Peptide-MHC binding affinity (the consensus method of the IEDB 2.17 software).	MHC I
NeoSplice (102) (2022)	RNA-seq (Alternative splicing)	Peptide-MHC binding affinity (Tool: NetMHCpan-4.0 [ANNs], NetMHCIIpan-3.2 [ANNs]. Input: peptide sequence and HLA type)	MHC I and MHC II

*ANN, artificial neural network.

These above-mentioned pipelines take into account the factors affecting antigen presentation and do not consider whether the presented antigen has the potential to elicit a T cell immune response. The advent of TIminer has led to a change in the way people think about developing neoantigen prediction. TIminer is a user-friendly computing framework that can perform different tumor immune genome analyses (88). In this pipeline, gene expression, immune infiltrates, and immunophenoscore are used to filter neoantigens. TIminer uses ANN-based NetMHCpan in the pipeline. TIminer is the first approach to introduce integrative immunogenomic analyses into neoantigen prediction, which increases the predicted number of neoantigens that can elicit an immune response. Kirchmair and Finotello (103) show us the usage of TIminer in identifying cancer neoantigens using public NGS data. It should to be noted that TIminer is limited to predict SNVs-derived neoantigens. Mupexi (92) is a program that identifies and prioritizes immunogenic peptides deriving from SNVs and indels using WES/WGS and RNA-Seq data. For the ranking of neoantigens, Mupexi uses a priority score that takes into account factors including the level of affinity between the mutant and the normal peptide, the allele frequency and gene expression level of the mutant. ANN-based NetMHCpan was also used for peptide-MHC binding calculation. Compared with pVACseq, MuPeXI offers more information and prioritizes the neoantigens, guiding the user to select the neoantigens that elicit a T cell response. However, the priority scoring system was constructed using few data, which results in uncertainty about its true utility. In addition, MHC types, somatic mutations and gene expression levels were required to be offered by the user, which is inconvenient. NeoPredPipe (95), a high-throughput pipeline for neoantigen prediction which incorporates ANN-based NetMHCpan into its pipeline, also considers the immunogenicity of neoantigens. In this workflow, the possibility of the neoantigen being recognized by the TCR is evaluated, which makes it more likely that the predicted neoantigens elicit an immune response. However, the immune response induced by contact between neoantigens and TCR is influenced by a variety of factors that are not yet well understood. Therefore, neoantigen prediction based on the probability of recognition of neoantigens by TCR needs to continue to be mined and the prediction accuracy still needs to be improved. ASNEO established by Zhang et al. (100) to identify alternative splicing (AS) neoantigens using RNA-seq data also employed scoring schema. The pipeline uses NetMHCpan-4.0 to filter the mutated peptides. After a series of filtering steps, an immune score schema was introduced to evaluate the immunogenicity of identified neoantigens with features including the %rank of mutant and normal peptide-MHC affinity, the number of mismatches between the mutant peptide and normal peptide, the peptide cleavage probability, TAP transport efficiency, hydrophobicity score and T cell recognition score. The percentage of neoantigens validated is 0.49%, which is similar to other studies. However, the pipeline does not take matched normal gene expression data into account.

Although the accuracy of these pipelines has been improved to some extent by incorporating ML-based tools, there is still huge room for improvement in predicting tumor neoantigens. The potential strategies include: 1) increase the quality of training data and enlarge the quantity of the data to ensure the data was collected from subjects from an unbiased ancestry and ethnicity background; 2) selectively use validated data with good validity and variety, covering a board range of cancer types and features for algorithm training; 3) employ advanced hardware and choose the right algorithm to learning the key features of neoantigen out of the big data required for neoantigen. Furthermore, most of the prediction pipelines are developed for SNVs and small indels derived neoantigens. Considering that neoantigens derived from other mutation types are more likely to elicit immune responses, we should pay more attention to studying the prediction pipelines of gene fusions, intron retentions and splice variant derived neoantigens.

### 4.2 ML-based neoantigen prediction utilizing immunogenic features

It should be noted that not all peptides presented by MHC molecule can induce T cell anti-tumor responses. Some features that used in scoring system have been proved to improve the accuracy of immunogenic neoantigen prediction. Other features, such as tumor abundance, peptide-MHC binding stability, polarity of amino acids, molecular size of peptides, entropy of peptides, and amino acid pairwise contact potentials also contribute to the immunogenicity of epitopes. In recent years, researchers integrated optimal potential immunogenic features with peptide-MHC binding and utilizing AI techniques to increase the accuracy of immunogenic neoantigen prediction. In this section, we summarize the recent new findings in the development of AI-based immunogenic neoantigen prediction pipelines.

As shown in **Table 2**, Neopepsee (104) is a first machine learning based neoantigen identification pipeline using NGS data. The machine learning classifiers used in immunogenic neoantigen prediction include support vector machine, random forest, locally weighted naive Bayes, and Gaussian naive Bayes. These models are constructed using the scores of 9 immunogenicity features covering IC_50_ of peptide-MHC I binding affinity, %rank of peptide-MHC I binding affinity, combined score, immunogenicity score, hydrophobicity score, polarity and charged score, differential agretopicity index (DAI), amino acid pairwise contact potentials (AAPPs), and similarity score to known peptides. The machine learning classifier is used to reduce the false positive rate that generate using only peptide-MHC binding affinity. The specificity is enhanced compared to conventional methods. Independent experimental data is employed to test the Neopepsee, which ascertain improved performance in melanoma and chronic lymphocytic leukemia. The Neopepsee improved classification power (0.48–0.56 of f-score) compared to conventional criteria (0.41–0.45 of f-score).

**Table 2:** Pipelines with ML models extracting binding affinity and immunogenicity feature.

Pipelines	Machine Learning Algorithm (and Scoring System)	Key Features	Data Input for each Feature
Neopepsee (104) (2018)	(1) Gaussian naive Bayes (2) Locally weighted naive Bayes (3) Random forest (4) Support vector machine	(1) IC_50_ (2) Rank (3) Combined score (4) Immunogenicity score (5) Hydrophobicity (6) Polarity and charged score (7) Differential agretopicity index (8) Amino acid pairwise contact potentials (9) Similarity	(1-2) Peptide sequence and HLA type (3-9) Peptide sequence
Machine-learning algorithm (105) (2019)	Gradient boosting model	(1) Valine at position 1 (2) Valine at the last position (3) Small amino acids at the last position (4) Basic amino acids of the reference sequence at the mutated position (5) Changes in the mutated position to a small amino acid (6) Lysine at relative site 1 (7) Presence of valine within the first 3 positions	Peptide sequence
pTuneos (14) (2019)	Random forest and scoring system	(1) Mutant pMHC affinity % rank (2) Normal pMHC affinity % rank (3) Sequence similarity between normal and mutant peptides (4) Peptide hydrophobicity score (5) T cell recognition probability of the pMHC complex	(1-2) Peptide sequence and HLA type (3-5) Peptide sequence
INeo-Epp (106) (2020)	Random forest	(1) The characteristics of 21 amino acids (2) Frequency score for immunogenic peptide (3) Peptide entropy (4) Rank (%) score (5) Differential agretopicity index	(1-3,5) Peptide sequence (4) Peptide sequence and HLA type
DeepAntigen (107) (2020)	Deep sparse learning	The 3D genome-related 2693 features	DNA and RNA-seq reads
TruNeo (108) (2020)	Deep learning and scoring system	(1) MHC binding (2) Proteasomal cleavage efficiency (3) TAP transport efficiency (4) Variant allele frequency (5) Expression abundance (6) Type of neoantigen	(1,3) Peptide sequence and HLA type (2) Peptide sequence (4) Mutate gene (5) RNA-seq reads (6) Mutate gene and affinity
DeepImmuno-CNN (109) (2021)	Convolutional neural network	Amino acid physicochemical features	Peptide sequence
Machine-learning algorithm (110) (2021)	Random forest	(1) Gene expression decile (2) Mutation present in RNA-seq (3) Mutant MHCflurry1.6 percentile rank (4) MHCflurry1.6 wild-type:mutant rank (5) C-term NetChop-3.1 score mutant (6) 20S NetChop-3.1 score mutant (7) NetMHCstabpan-1.0 prediction mutant (8) IEDB immunogenicity score mutant (9) TAP binding score mutant (10) T-cell contact residues hydrophobicity (11) T-cell contact (12) MHCflurry1.6 mutant processing score (13) MHCflurry1.6 mutant presentation score (14) Exome VAF decile	(1, 2) RNA-seq reads (3, 4, 7, 9, 12, 13) Peptide sequence and HLA type (5, 6, 8, 10, 11) Peptide sequence (14) WGS reads

Smith et al. (105) developed a gradient boosting machine learning-based algorithm to predict the immunogenicity of predicted peptide in neoantigen prediction pipeline. The neoantigen related peptide intrinsic biochemical characteristics including valine at position 1, valine at the last position, small amino acids at the last position, and so on (**Table 2**) were used to construct a gradient boosting model. The algorithm was then tested in two mouse tumor models and showed that the algorithm could predict antigens with potential therapeutic value. However, the mouse models only include two HLA types, which may miss the effect of HLA on immunogenicity.

The pTuneos (14) is a computational framework for prioritizing and selecting neoantigens from NGS data, which consists of four steps: (i) Data preprocessing. Sequencing quality control, alignment, quantification of the abundance of gene isoforms, variant calling, and HLA typing were performed in this step. (ii) Identification of candidate neoantigens. A list of candidates neoantigens was obtained by predicting the binding affinity of peptides-MHC. (iii) Training random forest model based on five non-redundant features related to the presentation and recognition of neoantigens (**Table 2**). The model initially screen neoantigens that can be presented by MHC I and recognized by TCRs. (iv) Neoantigen prioritization. A refined immunogenicity scoring schema used to evaluate the true immunogenicity of the identified neoantigens. The pTuneos was then verified effective using TIL infiltrating data sets, TCGA data sets, and Tumor immune checkpoint inhibitor treatment data sets. For evaluation of runtime, pTuneos was 20 times faster than Neopepsee and comparable to MuPeXI and pVAC-Seq. pTuneos obtained a higher performance than MuPeXI and Neopepsee when using naturally processed and presented neopeptides data. It is well known that the accuracy of machine learning models relies heavily on large amounts of data. However, the positive data (84 positive peptides) in training data and the testing dataset (21 peptides) were very few, which would affect the accuracy of the model. In addition, the immunogenic features used in this model were also few, which wouldn’t fully reflect immunogenicity.

INeo-Epp (106), a tool used to predict human immunogenic antigens and neoantigens, introduces HLA supertypes to improve the prediction accuracy of antigens presented by HLA. INeo-Epp combines the physical and chemical properties of amino acids of peptide, peptide structure characteristics, peptide entropy and peptide-MHC affinity %rank screening out 24 epitope immunogenicity related features and using supervised learning algorithm-random forest to predict the immunogenicity of epitopes. On this basis, a differential agretopicity index was added into features to identify neoantigens and the five-fold cross-validation showed good performance in predicting neoantigens. This method may increase the amount of true neoantigens that elicits an antitumor response. For future studies, the tool needs more data to perform an external verification of increased reliability.

Tang et al. (108) developed TruNeo, an integrated computational pipeline based on the deep learning model combined with scoring system, by taking into account the six main features that affect the prediction of neoantigens: peptide-MHC binding affinity, proteasome cleavage, antigen transporter transport efficiency, expression abundance, tumor heterogeneity, clonality and HLA LOH (loss of heterozygosity). This was done to best identify high confidence neoantigens with immunogenicity. The predictive performance of TruNeo and MHCflurry was compared using published literature and real patient data. TruNeo showed 52.63% of recall rate, which was 2.5 times higher than that obtained by MHCflurry (21.05%) using the published data. Furthermore, the positive rate of TruNeo (50%) was 2.5 times higher than MHCflurry (20%) (the lung cancer patient data). Both results showed that TruNeo exhibited more valuable predictive results than MHCflurry. However, only one real patient in a validation experiment is used for comparison between tools.

DeepImmuno-CNN (109), based on CNN, uses AAindex1 PCA encoding strategy to encode each amino acid sequence, overcoming the sparsity problems of one-hot encoding. Compared with DeepHLApan and IEDB, two commonly used immunogenicity identification approaches, the DeepImmuno has a better performance in predicting immunogenic neoantigens. DeepImmuno-CNN predicted 29 out of 35 (83%) immunogenic neoantigens, which was higher than IEDB (63%) and DeepHLApan (34%) using the tumor neoantigen dataset. Additionally, the DeepImmuno could help find residues that are vital for antigen recognition. It is noteworthy that the TCR sequence played an important role in the epitope recognition. However, this study and even most of studies not include the feature because of the shortage of the matched TCR sequencing data. In addition, this study only considerate the peptide sequence characteristics, which would bring bias to predict immunogenic neoantigens.

Due to the diversity and heterogeneity of T-cell immune responses, it is proposed that additional features can provide more precise information to accurately train a neoantigen-predicting model. It is believed that a systematic combination of multiple parameters would enhance the accuracy of established predictors. In addition to the features mentioned above, other features were proven to be associated with immune response, including the clonality of the neoantigen (111), CCR5 and CXCL13 expression (112, 113) and so on. These additional neoantigen parameters should be considered in training a complex model to strengthen the current neoantigen prediction algorithms.

### 4.3 Choice of the ML algorithms

As summarized in **Table 2**, the ML models employed in neoantigen-predicting pipelines are built by a verity types of ML algorithms, ranging from traditional ML algorithms (random forest, support vector machine etc.) to deep learning algorithms (such as convolutional neural network). Elaborately, the difference between deep learning algorithms and traditional ML or statistical analysis is that a deep learning technique learn the input data incrementally through multi-layered architecture. It is also named neural network because they are modeled after the human brain, allowing data to be processed between nodes in highly connected ways. The traditional approaches, including traditional machine learning and statistical modeling identify neoantigens through genomic NGS profiling, bioinformatics and mass spectrometry that always describe the classes of neoantigen with the sequencing variables and epitope/MHC binding affinity through a linear relationship. In contrast, the complexity and non-linear setting of the deep-learning callers determined that deep learning algorithms understand and fit better in terms of the non-linear association between NGS variables and neoantigen immunogenicity.

Deep learning become a more popular options mostly because several key advantages it displayed as comparing to traditional approaches. Firstly, compared to the old school bioinformatics algorithms, deep learning-based method could improve the degrees of feature extraction in neoantigen prediction, as neural networks outperform matrix-based methods in predicting peptide binding affinity. They are able to deal with less common peptides in variable lengths and structures and take into account the nonadditive effects, which may arise, e.g., when two amino acids compete for the same site in the peptide-binding groove of the MHC heterodimer (114). Secondly, deep learning-based method could improve the efficiency in identifying neoantigens. Deep learning algorithm is highly self-programming, which means that there is no need for manual supervision of the whole process, which therefore saves time and labor costs and therefore reduces human errors (115). Most importantly, the development of deep learning algorithms is accelerated and supported intensively by the modern computational tools and community. For instance, the 3D models of proteins that AlphaFold generates are far more accurate than before and thus would provide additional layer of information in an improved deep learning model representing a piece of information of neoantigen from an undercover angle (116). Merative, formerly IBM Watson Health, is an artificial intelligence assistant decision system providing sophisticated analyses to help identify the mutations responsible for cancer by combining cognitive computing and cloud computing with other advanced genomic sequencing technology (117). This type of tools is better defining the true mutation and the key variant information in oncogenic peptide, therefore is beneficial for neoantigen identification and model training.

Although the promise of deep learning algorisms is considerable, a long-standing concern about deep learning models is often referred to the “black boxes” issue with deep learning algorithms because they are so complex that human including the researchers who built them cannot straightforwardly interpret how the predictions were made. Lack of interpretability in deep learning-based models does not help researchers to understand the underlying scientific associations between neoantigen immunogenicity with the immunogenic mutations. One of the potential solutions is to build an explainable deep learning approach through techniques replacing deep learning black-box models with simpler interpretable models that can explain the features and models to human (118, 119). A following question, once an explainable deep learning model is available, is how to achieve an explainability for different types of predictors, considering the tradeoff between the predicting accuracy and transparency associated with scientific understanding, and to develop and deploy trustworthy deep learning-based approaches that meet healthcare objectives.

## 5 Discussion

The advances of AI in biomedicine promote the accuracy of neoantigen prediction *in silico*, which is reflected in the improvement of the accuracy of AI-based methods as compared to other strategies-based methods (**Table S5**). With the popularization of large-scale genome and transcriptome sequencing, as well as the development of single-cell sequencing technology, more and more data are generated in biomedicine. Employing AI to train existing cancer clinical data, including tissue slice images, sequencing data, clinical data, etc., makes it possible to find deep underlying commonalities in the big data, which help us understand the unique characteristics of different cancer cells. For example, Reiman et al. (120) used neural network models to accurately characterize the tumor immune microenvironment of solid tumors in the large intestine, breast, lung, and pancreas by integrating RNA-Seq and imaging data, which is crucial for determining the patient’s response to cancer immunotherapy. AIDeveloper, a deep learning software, was developed by Kräter et al. (121) to classify image without the required for programming. In addition, combination of AI and microfluidics can bring convenience for biotechnology study. Almost all aspects of biomedical fields, including but not limited to diagnosis, personalized medicine, and treatment of oncology would be benefited from this combination (122). For example, clinical decision-makings such as patient screening for immunotherapy and the prediction of response to treatment, were improved significantly by advanced AI technique (123, 124). Through the AI analysis of genome mutations, transcriptomes and other data, we can understand why differences develop among different individuals. This will provide a reference for future treatment methods. The application of AI to genomics, transcriptomics and proteomics makes it the greatest asset in neoantigen prediction. For example, AI can be used for mutation identification, proteasomal cleavage site predictions and TAP transport efficiency predictions during neoantigen processing and presentation, assessment of peptide-MHC binding affinity, and prediction of the immunogenicity of neoantigens. Compared with bioinformatic tools, the number of predicted effective neoantigens using AI model has increased dramatically, which has greatly boosted immunotherapy (**Table S5**).

Major neoantigen prediction methods mainly focus on peptide-MHC binding affinity. However, most predicted candidate neoantigens that presented by MHC molecules will not provoke T cell immune responses. In order to solve this problem, some immunogenic features are used as filters in the workflow of neoantigen prediction and AI models are being established for identifying the immunogenicity of epitopes that using a set of immunogenicity related features. In 2018, neopepsee was first developed to predict immunogenic neoantigens using machine learning model. In the following years, more and more methods based on machine learning models have been developed to predict the immunogenicity of neoantigens to improve the prediction accuracy. It is worth emphasizing that the models are very important for the prediction accuracy of neoantigens not only in the step of predicting the immunogenicity but also in the most of neoantigen prediction steps from the variant calling to the prediction of TCR-pMHC binding. And the models used in the steps of neoantigen prediction mainly including conventional machine learning (such as random forest) and deep learning including DNN, ANN, and CNN. Each model has its own advantages in the corresponding application. For example, Zhou et al. (14) choose the random forest (testing AUC of 0.833) rather than eXtreme Gradient Boosting (testing AUC of 0.654) in the study due to the high accuracy rate of random forest. Generally, conventional machine learning models require structured data, however, deep learning models are better at working with unstructured data. Therefore, we can see from the above-mentioned sections that deep learning models are more applied to the development of methods that use unstructured data as input including variant callers, peptide-MHC binding predictors, TCR- pMHC binding predictors and immunogenicity of peptide predictors. And conventional machine learning models such as random forest are applied to development of methods that use unstructured data as input (**Table 2**). It is important to select an optimal model for our study. Li et al. (109) performed an evaluation for 5 traditional machine learning including K-Nearest Neighbors, Support Vector Machine, ElasticNet, AdaBoost, and Random Forest and 3 deep learning models including ResNet, CNN, and GNN with prior validated data. Finally, the CNN model of the best performance was selected to use in the study. Hence, it is a necessary step to evaluate the available ML models and select best one before conducting study.

It should be noted that the quantity and quality of features and data are the most critical factors for machine learning models. Currently, the immunogenicity related features of neoantigens are not exactly sure. Researchers used different features to training their models. However, there are several features that are considered to be related to the immunogenicity of neoantigens. Peptide-MHC binding affinity was considered as an excellent feature for prediction of immunogenicity. Bjerregaard et al. found that compared to non-immunogenic peptides, immunogenic neopeptide have a stronger binding affinity on MHC molecule (78). In addition, combination with the non-mutated peptide-MHC binding affinity can also improve the accuracy of neoantigen prediction (125). Previous studies have shown that the binding stability of the peptide-MHC was an important feature for prediction of immunogenicity (126). However, Koşaloğlu-Yalçın et al. (125) indicated that compared to binding affinity prediction alone, binding stability predictions alone and combination with binding affinity predictions did not improve the prediction accuracy of immunogenic neoepitope. Tumor Neoantigen Selection Alliance (TESLA) showed that binding stability of peptide-MHC was related with peptide immunogenicity (1). And Gartner et al. (110) found that the stability scores of positive short peptides were significantly higher than negative short peptides and combination with binding stability score and binding affinity score could improve the accuracy of neoepitope prediction. Therefore, there was controversial on the relationship between a binding stability of peptide-MHC and immunogenicity. The tumor abundance, a key presentation-related factor, was also deemed to be associated with neoantigen immunogenicity by TESLA (1). It has been reported that the DAI was a better feature for prediction of immunogenic neoepitopes (127). However, Koşaloğlu-Yalçın found that DAI performed worse than only binding affinity that predicted by NetMHCpan (125). Several studies indicated that sequence similarity (78) and the hydrophobicity of amino acids at TCR contact residues (79) were important features of immunogenicity. Other important experimental features about HLA molecules, peptides, and TCRs that can represent the immune response relationship should be discovered to enable better neoantigen prediction. Studies have shown that anti-tumor responses were mediated by T cells that target cancer cells by recognizing neoantigens presented on HLA molecules (83, 128). Therefore, the interaction between TCRs and their tumor epitopes is essential for the anti-tumor immune response (129). Most T cells with tumor-specific TCRs showed an exhausted state that reduction of function following prolonged exposure to antigens (130). Similarly, it has been shown that neoantigen-specific TILs were largely in a CD39^+^CD69^+^ differentiated state, which may lead to no response to TILs-based adoptive T cell therapy (131). However, complete responders of TILs-based adoptive T cell therapy retained a CD39^-^ subpopulation of stem cell-like neoantigen-specific TIL cells. Together, incorporating T cell phenotype, such as T cell dysfunctional and progenitor states would allow to effectively predict immunogenic neoantigens for cancer immunotherapy. Although there are likely difficult to incorporate these features, the future endeavors can address such challenge with the advances in biotechnology and machine learning. Although many features were found to be related with peptide immunogenicity, correlation between features makes features to be redundant, which would make the AI models to be fitted. To overcome this, some ML models especially random forest model was selected to select features due to the ability that random forest model could find the correlations between features, which provides unbiased method to select important features for prediction of neoantigens (106, 110).

Learning from a large amount of data and identifying patterns that we were not previously aware of is the biggest advantage of ML. However, due to various reasons, such as inconsistent business purposes or related to personal privacy laws, some stakeholders are reluctant to exchange data with each other. Insufficient data is one of the main reasons that the effectiveness of AI may be limited, especially considering there are currently only a small number of public databases available, such as IEDB, TSNAdb v1.0, and CAD v1.0. The Immune Epitope Database (IEDB) is a free resource funded by the National Institute of Allergy and Infectious Diseases (NIAID) (132). It documents experimental data on antibodies and T-cell epitopes studied in human, non-human primate, and other animal species in the context of infectious disease, allergy, autoimmunity, and transplantation. IEDB is currently the most used database to provide epitope data for development of methods for neoantigen prediction. And IEDB also provide neoantigen prediction tools for researchers. However, the most of epitope data were from bacteria or viruses, which may lead to bias for the human neoantigen prediction (133). TSNAdb v1.0 (134), a database of tumor neoantigens, collected somatic mutation and HLA alleles information from TCGA and TCIA databases for a total of 7748 tumor samples from 16 tumors. Two software, NetMHCpan v2.8 and NetMHCpan v4.0, respectively, were used to predict the binding affinity of the mutated peptides and HLA. It should be noted that this database does not include the experimental validated neoantigen data. The Cancer Antigens Database (CAD v1.0) is developed by Yu et al. (135) in 2022 year to provide a platform for cancer antigen especially neoantigens related methods development. In CAD v1.0, verified tumor antigens and relevant peptide information were collected. However, the neoantigen data may from many ethnicities, which also bring bias for prediction of neoantigen. In addition, data imbalance problem also influences the accuracy of the AI model though there are some methods to reduce the impact bring by data imbalance. For example, Zhou et al. (14) used 84 positive data and 2107 negative data to train random forest model, which was significantly imbalanced.

Although there are many obstacles on the neoantigen prediction, the puzzle of neoantigen prediction will eventually be solved with the ever-advancing sequencing technology, mass spectrometry technique, laboratory biotechnology and AI models, more precise immunogenic features, and more available data. In the future, more attention on the immunity response induced through interaction of tumor and T cell. At present, our understanding of the intrinsic tumor-specific immune responses is restricted, which limited identification of new immunogenic neoantigens. The properties of neoantigen-specific T cells, such as T cell phenotypes, T cell dysfunctional and progenitor states, are proven to impact anti-tumor response, and thus are expected to serves as additional source of features for neoantigen prediction. With the development of single cell sequencing, the single cell mapping of neoantigen-reactive T cells would allow data mining of new gene phenotype and molecular characteristics of T cells (136). In addition to coding region proteins, more studies are expected to focus on epitopes that are not belong to encoding region given the influence of these peptides to the anti-tumor immune response is largely discovered. Compared with personalized neoantigens, shared neoantigens are the best option but more difficult to identify. The shared neoantigen is derived from a driver mutation in an oncogene or other hotspot mutation in the genome. Study showed that a proportion of patients with epithelial tumors have antigen-specific T cell responses to TP53 hotspot mutations (137). A major advantage of shared neoantigens is that they were proven to be successfully applied to clinical care in short time. Therefore, it is expected the predictive accuracy of shared neoantigens would be greatly improved in the future. Combination with the big data and AI to analyze the characteristics of real neoantigens, thereby improving predictive accuracy and better exploiting their role in immunotherapy such as a neoantigen-based vaccine, cell therapy, a combination of vaccines and other immunotherapies or traditional therapies.

## 6 Conclusion

AI-based platforms for neoantigen prediction have received increasing attention in recent years. The introduction of AI models solves problems that bioinformatics methods cannot, such as assessing the ability of a predicted neoantigen to elicit an immune response. Creating scores to refine predicted candidate neoantigens improves the accuracy of neoantigen prediction pipelines. In this manuscript, we described a neoantigen identification workflow and pipeline. We highlighted AI-based tools and models in the process from data processing to final neoantigen determination. Although several computational and experimental approaches are being used for research and clinical trials, there is still an urgent need to further optimize these methods and develop new accurate pipelines. The current methods mainly focus on peptide-MHC binding affinity, which lead to few predicted candidate neoantigens that can induce T cell response by *in vitro* or *in vivo* experiments. To address this problem, efforts are now underway to develop new AI-based algorithms to predict immunogenic neoantigens. Our knowledge of the features of immunogenic neoantigens and the mechanism of TCR recognition of tumor antigens has considerable room for improvement. Despite the existing challenges of neoantigen prediction, the future of neoantigen-based immunotherapy is bright, largely due to advancements in AI.

## Author contributions

YC and RC: Conceptualization, Investigation, Data curation, Writing - Original draft, Writing - Review & Editing, Visualization. SG and WL: Investigation, Data curation. YL, GS, MS, MJ: Data curation. CJ: Review. XZ: Writing and Revision. All authors contributed to the article and approved the submitted version.
