# Supplementary material for: Artificial intelligence applied in neoantigen identification facilitates personalized cancer immunotherapy
Source: Front Oncol. 2023 Jan 9;12:1054231. doi: 10.3389/fonc.2022.1054231 (PMC9868469; doi:10.3389/fonc.2022.1054231)
Supplement: Supplementary file 1 [file DataSheet_1.docx]

Supplementary Material

Artificial Intelligence Applied in Neoantigen Identification Facilities Personalized Cancer Immunotherapy

Yu Cai^1†^, Rui Chen^1†^, Shenghan Gao^1^, Wenqing Li^1^, Yuru Liu^1^, Guodong Su^1^, Mingming Song^1^, Mengju Jiang^1^, Chao Jiang^2*^, Xi Zhang^1*^

^1^School of Medicine, Northwest University, Xi’an, Shaanxi 710069, China

^2^ Department of Neurology, The Second Affiliated Hospital of Xi'an Medical University, Xi’an, Shaanxi 710038, China

†These authors have contributed equally to this work

*** Correspondence:**Corresponding Author
jiangbeyound0818@163.com (Chao Jiang)

[xzhang19@nwu.edu.cn](mailto:xzhang19@nwu.edu.cn) (Xi Zhang)

**Table S1.** Sources of data used in development of ML-based methods for neoantigen prediction.

| **Section** | **ML-based Approach** | **Description of Source Data** |
| --- | --- | --- |
| Variant Calling | DeepVariant (1) | Labeled images, including RGB pileup images with known genotypes encoded by the reference and read bases, quality scores, and other read features. |
|  | Cerebro (2) | Normal peripheral blood DNA sample in which exome regions were captured and sequenced twice using NGS methods. Over 30,000 somatic variants at mutant allele frequencies from 1.5% to 100%. |
|  | NeuSomatic (3) | Contrived virtual tumor and normal pair data derived from data generated from “normal” cell line NA12877; real tumor-normal pair data built from CLL1 chronic lymphocytic leukemia data and the COLO-8 immortal metastatic malignant melanoma cell line data. |
| Peptide-MHC binding prediction | NetMHCpan 4.0 (4) | Quantitative binding data and MS derived MHC eluted ligands collected from Immune Epitope Database (IEDB database). |
|  | MHCflurry 2.0 (5) | MS entries and affinity measurements including peptide sequence (length 8-15) and MHC class I allele. |
|  | ACME (6) | MHC class I binding affinity dataset (IEDB database) including peptide sequence and MHC class allele. |
|  | IConMHC (7) | Binding data (IEDB database) including peptide sequence and MHC class allele. MHC-I protein sequences. |
|  | MHCnuggets (8) | Chemical binding affinity measurements (IEDB database) for peptide-allele pairs covering both class I alleles class II alleles. |
| TCR-pMHC binding prediction | ERGO (9) | TCR-binding peptides from McPAS-TCR and VDJdb databases including CDR3 sequence of each CD4 and CD8 T cell receptor and matched peptide sequence. |
|  | NetTCR-2.0 (10) | Positive data set: peptide-CDR3β binding pairs collected from IEDB.  Negative data set: nonbinding peptide-CDR3β pairs derived from 10X Genomics Chromium Single Cell Immune Profiling of four donors. |
|  | pMTnet (11) | TCR sequence (CDR3β), antigen sequence, and class I MHC allele from high-throughput experiments. |
| Immunogenic neoantigens prediction | Immunogenicity classification (12) | Positive dataset: 311 known immunogenic epitopes, their corresponding HLA alleles, and their corresponding wild-type.  Negative dataset: 14633 mutant peptides that would not lead to an immunogenic response. The HLA allele for negative dataset was randomly selected from HLA alleles of the positive set. |
|  | Immunogenicity prediction of MHC I epitopes (13) | Total immunogenic epitope data (n=210) of MHC I were from ELISpot screening. And 141 peptides were used for exploration (training and test) and 69 peptides were used for validation. Total 68 immunogenic MHC II epitopes were also obtained from ELISpot screening. |
|  | Pre&RecNeo (14) | The training dataset included 84 positive peptides that could elicit a T cell response and 2107 negative peptides. The testing dataset (21 peptides) was obtained from Carreno et al. (15). Nine of 21 tested peptides were immunogenic. |
|  | INeo-Epp (16) | The neoantigen data that were collected from 11 publications (17) and IEDB mutational epitopes. The datasets included nonimmunogenic neoepitopes and immunogenic neoepitopes, as well as their corresponding HLA alleles and wild-type. |
|  | MMP (mutated minimal peptides) model (18) | 120 class I mutated minimal peptides and their corresponding HLA restriction elements, as well as their wild-type. |

**Table S2.** Variant calling tools and its pros and cons.

| **Tool (Year)** | **Mutation** | **Pros** | **Cons** |
| --- | --- | --- | --- |
| VarScan2 (19)  (2012) | SNV/Indel | (1) VarScan2 can simultaneous detection of germline variants, somatic mutations, LOH, and SCNAs using exome sequence data from matched tumor and normal samples. (2) VarScan2 exploits the digital nature of massively parallel sequence data to detect small but significant differences between normal and tumor samples. | The default VarScan2 settings makes it unable to call somatic SNVs of low allelic fraction. |
| SomaticSniper (20)  (2012) | SNV | For high-allelic-fraction somatic SNVs, SomaticSniper perform better in terms of sensitivity in both amplicon sequencing and exome sequencing data. (2) SomaticSniper achieves the higher sensitivity in the 100% ‘pure tumor’-normal match. | The rapid decline of detection power that occurs when the normal sample contains tumor cells. |
| FaSD (21)  (2012) | SNV | FaSD makes accurate SNP calls even at loci with low-depth reads, with intermediate sequencing quality, and at the two ends of the read, compared with other programs that failed to make SNP calls. | FaSD can only identify SNPs. |
| Mutect2 (22)  (2013) | SNV/Indel | (1) Mutect2 can call somatic mutations in tumor-only mode. (2) MuTect2 can robustly detect somatic SNVs at low allelic fraction. | The calling results require careful filtering for false positives due to the deficiency of corresponding germline information. |
| Shimmer (23)  (2013) | SNV | Shimmer produces somatic SNVs predictions with significantly higher sensitivity and accuracy than other available software when run on highly contaminated or heterogeneous samples, and it gives comparable sensitivity and accuracy when run on samples of high purity. | Shimmer is less sensitive at lower depths of sequencing. |
| EBCall (24)  (2013) | SNV/Indel | EBCall can effectively detect a series of somatic mutations that have allele frequencies of <10% with a high degree of accuracy, thereby identifying subclonal structures of cancer cells that cannot otherwise be found. | The tool is confined application to exome sequencing data. |
| RADIA (25)  (2014) | SNV | (1) The inclusion of the RNA increases the power to detect somatic mutations, especially at low DNA allelic frequencies. (2) By integrating an individual’s DNA and RNA, RADIA is able to detect mutations that would otherwise be missed by traditional algorithms that examine only the DNA. | (1) Only expressed alleles can be evaluated, which reduces the number of genes that can be assessed. (2) several classes of mutations, such as the introduction of premature stop codons that lead to nonsense mediated decay, cannot be verified. |
| VarDict (26)  (2016) | SNV/Indel/MNV | (1) VarDict can perform local realignment over indels on the fly for more accurate allele frequencies of indels. (2) VarDict can rescue softly clipped reads to identify indels not present in the alignment or support existing indels. (3) VarDict has very efficient memory management and memory usage is linear to the region of interest, not the depth. (4) VarDict can handle ultra-deep sequencing and the performance is only linear to the depth. | In order to increase the specificity, VarDict currently heavily relies on split reads, coupled with discordant mate pairs to detect SVs at precise breakpoints. This does limit its sensitivity in lower coverage WGS where split reads are less likely to be sequenced and aligned. |
| Lancet (27)  (2018) | SNV/Indel | (1) Lancet shows high precision when calling somatic mutations and provides robust calls across data. (2) In addition to being used as a genome-wide analysis tool, Lancet can be used interactively to call variants and render colored de Bruijn graphs at small genomic regions of interest. (3) Lancet can reliably detect deletions up to 400 base pairs in length and insertions shorter than 200 bp. | (1) Its sensitivity is reduced for longer mutations and structural variations, especially in the case of novel insertions, tandem duplications, and mobile elements whose reads could be either unmapped or mapped to a different copy of the repeat. (2) Due to its pure local-assembly strategy, Lancet currently has longer runtimes compared to alignment-based methods. |
| Strelka2 (28)  (2018) | SNV/Indel | (1) Strelka2 shows higher sensitivity in detecting low-allelic-fraction SNVs in amplicon sequencing reads, with good specificity. (2) Strelka2 can robustly detect somatic SNVs at low allelic fraction. | Context-sensitive base-calling errors would affect SNV calling. |
| DeepVariant (1)  (2018) | SNV/Indel | (1) It is the first to apply neural networks to the detection of biological sequence variants. (2) DeepVariant transforms the huge workload of the splicing problem (splicing of HTS fragmentation results into complete gene sequences) into a typical image classification problem. | The open source DeepVariant takes a very long time. |
| Cerebro (2)  (2018) | SNV/small indel | Cerebro is totally automated without the need for expert to supervise sequence data. | Cerebro is unsustainable for widespread NGS analyses. |
| ABRA2 (29)  (2019) | SNV/Indel | (1) ABRA2 results in broad improvements to variant calling accuracy across a wide range of post-processing workflows including whole genomes, targeted exomes and transcriptome sequencing. (2) ABRA2 was designed with RNA-Seq in mind and does not require special processing to treat RNA-Seq data as if it were DNA. (3) By directly making use of splice junction information, ABRA2 is able to achieve greatly improved accuracy over other methods. | Alignment of each contig to each putative transcript is currently the computational bottleneck for ABRA2 |
| NeuSomatic (3)  (2019) | SNV/Indel | (1) High accuracy. (2) Avoiding missed tests. (3) Achieving the same level of accuracy as previous methods also requires significantly fewer sequencing data, which can reduce the cost of the assay. (4) Methodologically, the method is innovative in that it uses the output and intermediate results of other assays as input, thus allowing the integration of multiple methods under one framework. | Low training data |
| Pindel (30)  (2009) | structural variants | The pattern growth method is very efficient in searching for deletions up to 10 kb long, taking only 4.5 h on a single CPU to process data derived from ∼40× coverage paired-end reads of human genome. | (1) Pindel only considers perfect matching and mismatch is not allowed. (2) As a consequence, SNPs or sequencing base error in the regions of anchor or indel points may lead to the miss of true positives because there might be insufficient supporting reads. (3) Pindel does need read mapping and this process is the main bulk of computational CPU time and memory usage |
| Lumpy (31)  (2014) | structural variants | Lumpy yields improved sensitivity, especially when SV signal is reduced owing to either low coverage data or low intra-sample variant allele frequency. | (1) Raw read-depth data was not used. (2) Substantial improvements to sensitivity may require more comprehensive and accurate SV catalogs than are currently available. |
| Manta (32)  (2016) | structural variants/Indels | (1) Manta can be used for rapid germline and somatic analysis, calling structural variants, medium-sized indels and large insertions on standard computer hardware in less than a tenth of the time that comparable methods require to identify only subsets of these variant types. (2) Manta can discover and score variants based on supporting paired and split-read evidence. (3) Manta consistently assembles a higher fraction of its calls to base-pair resolution, allowing for improved downstream annotation and analysis of clinical significance. | Manta is only suitable for high parallelization on individual or small sets of samples. |
| EagleC (33)  (2022) | structural variants | (1) EagleC can predict a full range of SVs at high resolution. (2) EagleC can uniquely capture a set of fusion genes that are missed by whole-genome sequencing or nanopore. (3) EagleC can effectively captures SVs in other chromatin interaction platforms. (4) EagleC can be applied to single-cell Hi-C and used to study the SV heterogeneity in primary tumors. | Because the data collected in this study had various sequencing depths and quality, the tool is limited at the 5-kb resolution and predicted SVs with a minimum size of 35 kb. |
| deFuse (34)  (2011) | gene fusion | deFuse considers all alignments and all possible locations for fusion boundaries. As a result, deFuse is able to identify fusion sequences with demonstrably better sensitivity than previous approaches. | (1) The main limitation of deFuse is the requirement of at least five discordant read pairs to nominate a gene fusion to the adaboost classifier. This will certainly miss fusions that have very low expression and may result in insensitivity to fusions from RNA-Seq datasets with minimal sequence generation. (2) deFuse rely on an annotated set of genes. |
| SOAPfuse (35)  (2013) | gene fusion | Compared with other released tools, SOAPfuse achieves higher detection efficiency and consumed fewer computing resources. | (1) In the simulated dataset, SOAPfuse missed three fusion transcripts. (2) The program had some difficulties detecting fusion transcripts from gene pairs having highly similar sequences, and fusion transcripts involving short transcripts of long genes. |
| JAFFA (36)  (2015) | gene fusion | (1) JAFFA is a sensitive fusion detection method that outperforms other methods with reads of 100 bp or greater. (2) JAFFA compares a cancer transcriptome to the reference transcriptome, rather than the genome. | (1) JAFFA’s RAM utilization in assembly mode was not constant, but scaled with the input reads due to the de novo assembly. (2) JAFFA’s Direct mode is only suitable for reads of 100 bp. |
| GFusion (37)  (2017) | gene fusion | GFusion performs multiple alignments and strict filtering algorithm to improve sensitivity and reduce the false positive rate. | GFusion filters out false fusions through a series of filtering steps. |
| Fusion-Bloom (38)  (2020) | gene fusion | Fusion-Bloom leverages recent developments in de novo transcriptome assembly and assembly-based structural variant calling technologies. | The tool is suitable for short reads. |
| MiSplice (39)  (2018) | alternative splicing | (1) The software can be easily deployed in a higher performance computing framework. (2) The software can be readily applied to datasets of thousands of samples. (3) SplAdder is a convenient one-stop-shop that provides all analysis within a single pipeline. | High coverage results in lower performance, most likely due to more false positives in the predicted set. |
| MMsplice (40)  (2019) | alternative splicing | (1) All MMSplice modules and models are shared in the model repository Kipoi, which should allow other computational biologists to improve individual modules or to flexibly include modules into their own models. (2) The models outperformed models based on the reference genome and natural variations and was only matched by models based on perturbation assays. | (1) Splicing is known to be tissue-specific, while this model is not. (2) Their exon and intron modules have only one convolutional layer, which is not enough to learn complex interaction effects of splicing regulatory elements. |
| iRead (41)  (2020) | alternative splicing | (1) iREAD detects retained introns through analyzing both splice junction reads and intron expression level that considers all the intronic reads to increase confidence. (2) iREAD is suitable for both single-end and paired-end sequencing data. | iREAD may fail when the intron has low absolute expression (FPKM) though the relative expression of intron-retained transcripts (i.e. IRratio) can be high. |
| RES-Scanner (42)  (2016) | RNA editing | (1) RES-Scanner is capable of calling homozygous genotypes reliably from samples with any ploidy number, including samples from a pool of multiple individuals. (2) RES-Scanner implements binomial tests to rigorously distinguish RNA-editing sites from sequencing errors by assigning a p-value to each RNA-editing candidate. (3) RES-Scanner provides a complete pipeline from raw sequencing reads to final editing sites, which should be especially valuable to users who have limited experience in bioinformatics or are working with non-model species with no prior knowledge of the optimal mapping strategy. | It run more slowly than GIREMI, it does not work for non-diploid samples or species with limited SNP information (e.g. most non-model species). In fact, the RES-Scanner is designed to run multiple samples in parallel, so that an increase in sample numbers will not greatly affect the overall runtime if sufficient computing nodes are available. |
| RNAEditor (43)  (2017) | RNA editing | (1) RNAEditor seems to detect more specific editing sites. (2) The concept of editing islands is introduced, which can be detected through RNAEditor. These editing islands help highlight important regions in where editing events occur frequently. | (1) REDItools outperforms RNAEditor in terms of computational time. (2) It uses BWA as an aligner, which is not developed exclusively for RNA-seq data. Thus, splice junction reads might not be detected in the current workflow of RNAEditor. |
| DeepRed (44)  (2018) | RNA editing | (1) DeepRed identifies RNA editing from primitive RNA sequences without prior-knowledge-based filtering steps or genomic annotations. (2) DeepRed offers better prediction accuracy and computational efficiency than current methods with large-scale, mass RNA-seq data. (3) It may decipher the hidden principles behind RNA editing, making editing detection convenient and effective. (4) DeepRed can be applied to any RNA-seq dataset without restrictions. (5) DeepRed is capable of learning the general features of RNA editing across multiple samples. | It may not be sensitive to the recognition of RNA editing sites unique to a single sample. |

**Table S3.** HLA typing tools *in silico*.

| **Tool** | **Data type** | **MHC class** | **Resolution** | **Year** |
| --- | --- | --- | --- | --- |
| Seq2HLA (45) | RNA-seq | I and II | Two digits | 2012 |
| SoapHLA (46) | WGS | I and II | Four digits | 2013 |
| HLAforest (47) | RNA-seq | I and II | Eight digits | 2013 |
| Optitype (48) | WES/WGS/RNA-seq | I | Four digits | 2014 |
| Athlates (49, 50) | WES | I and II | Four digits | 2013, 2015 |
| Polysolver (50) | WES | I | Eight digits | 2015 |
| HLAMiner (51, 52) | WES/WGS/RNA-seq | I and II | Four digits | 2015, 2012 |
| HLAreporter (52) | WES | I and II | Six digits | 2015 |
| HLA-VBSeq (53) | WGS | I and II | Eight digits | 2015 |
| HLAscan (54) | WES/WGS | I and II | Four digits | 2017 |
| HLAProfiler (55) | RNA-seq | I and II | Six digits | 2017 |
| xHLA (56) | WGS/WES | I and II | Four digits | 2017 |
| HLA-HD (57) | WES/WGS/RNA-seq | I and II | Six digits | 2017 |
| Kourami (58) | WGS/WES | I and II | Six digits | 2018 |
| PHLAT (59) | WES/WGS/RNA-seq | I and II | Six digits | 2018 |
| HLA*LA (60) | WGS/WES | I and II | Eight digits | 2018 |
| ArcasHLA (61) | RNA-seq | I and II | Six digits | 2020 |

**Table S4.** Tools for peptide-MHC binding prediction.

| **Tool** | **MHC class** | **Year** | **Tool** | **MHC class** | **Year** |
| --- | --- | --- | --- | --- | --- |
| SMM (62) | I | 2007 | TEPITOPE (63) | II | 1999 |
| SMMPMBEC (64) | I | 2009 | ProPred (65) | II | 2001 |
| Pickpocket (66) | I | 2009 | RANKPEP (67) | II | 2002 |
| NetMHCcons (68) | I | 2012 | SVRMHC (69) | II | 2006 |
| NetMHC4 (70) | I | 2016 | SMMAlign (62) | II | 2007 |
| NetMHCpan4 (4) | I | 2017 | Comblib (71) | II | 2008 |
| MHCnuggets (72) | I | 2017 | NNAlign (73) | II | 2011 |
| MixMHCpred (74) | I | 2017 | MultiRTA (75) | II | 2010 |
| ConvMHC (76) | I | 2017 | TEPITOPEpan (77) | II | 2012 |
| MHCflurry (78) | I | 2018 | OWA-PSSM (79) | II | 2013 |
| MHCSeqNet (80) | I | 2018 | NetMHCIIpan (81) | II | 2018 |
| ACME (6) | I | 2019 | Trans-Allelic Model (82) | II | 2018 |
| IConMHC (7) | I | 2020 | MixMHC2pred (83) | II | 2019 |
| Anthem (84) | I | 2021 | MARIA (85) | II | 2019 |
| RBM-MHC (86) | I | 2021 | NeonMHC (87) | II | 2019 |
| APPM (88) | I | 2021 | MHCnuggets (8) | I and II | 2020 |
| DeepNetBim (89) | I | 2021 | DeepSeqPanII (90) | II | 2022 |
| MHCRoBERTa (91) | I | 2022 |  |  |  |

**References**

1. Poplin R, Chang PC, Alexander D, Schwartz S, Colthurst T, Ku A, et al. A Universal Snp and Small-Indel Variant Caller Using Deep Neural Networks. *Nat Biotechnol* (2018) 36(10):983-7. Epub 2018/09/25. doi: 10.1038/nbt.4235.

2. Wood DE, White JR, Georgiadis A, Van Emburgh B, Parpart-Li S, Mitchell J, et al. A Machine Learning Approach for Somatic Mutation Discovery. *Science translational medicine* (2018) 10(457). Epub 2018/09/07. doi: 10.1126/scitranslmed.aar7939.

3. Sahraeian SME, Liu R, Lau B, Podesta K, Mohiyuddin M, Lam HYK. Deep Convolutional Neural Networks for Accurate Somatic Mutation Detection. *Nat Commun* (2019) 10(1):1041. Epub 2019/03/06. doi: 10.1038/s41467-019-09027-x.

4. Jurtz V, Paul S, Andreatta M, Marcatili P, Peters B, Nielsen M. Netmhcpan-4.0: Improved Peptide-Mhc Class I Interaction Predictions Integrating Eluted Ligand and Peptide Binding Affinity Data. *Journal of immunology (Baltimore, Md : 1950)* (2017) 199(9):3360-8. Epub 2017/10/06. doi: 10.4049/jimmunol.1700893.

5. O'Donnell TJ, Rubinsteyn A, Laserson U. Mhcflurry 2.0: Improved Pan-Allele Prediction of Mhc Class I-Presented Peptides by Incorporating Antigen Processing. *Cell systems* (2020) 11(1):42-8.e7. Epub 2020/07/28. doi: 10.1016/j.cels.2020.06.010.

6. Hu Y, Wang Z, Hu H, Wan F, Chen L, Xiong Y, et al. Acme: Pan-Specific Peptide-Mhc Class I Binding Prediction through Attention-Based Deep Neural Networks. *Bioinformatics (Oxford, England)* (2019) 35(23):4946-54. Epub 2019/05/24. doi: 10.1093/bioinformatics/btz427.

7. Pei B, Hsu YH. Iconmhc: A Deep Learning Convolutional Neural Network Model to Predict Peptide and Mhc-I Binding Affinity. *Immunogenetics* (2020) 72(5):295-304. Epub 2020/06/25. doi: 10.1007/s00251-020-01163-9.

8. Shao XM, Bhattacharya R, Huang J, Sivakumar IKA, Tokheim C, Zheng L, et al. High-Throughput Prediction of Mhc Class I and Ii Neoantigens with Mhcnuggets. *Cancer immunology research* (2020) 8(3):396-408. Epub 2019/12/25. doi: 10.1158/2326-6066.Cir-19-0464.

9. Springer I, Besser H, Tickotsky-Moskovitz N, Dvorkin S, Louzoun Y. Prediction of Specific Tcr-Peptide Binding from Large Dictionaries of Tcr-Peptide Pairs. *Frontiers in immunology* (2020) 11:1803. Epub 2020/09/29. doi: 10.3389/fimmu.2020.01803.

10. Montemurro A, Schuster V, Povlsen HR, Bentzen AK, Jurtz V, Chronister WD, et al. Nettcr-2.0 Enables Accurate Prediction of Tcr-Peptide Binding by Using Paired Tcrα and Β Sequence Data. *Communications biology* (2021) 4(1):1060. Epub 2021/09/12. doi: 10.1038/s42003-021-02610-3.

11. Lu T, Zhang Z, Zhu J, Wang Y, Jiang P, Xiao X, et al. Deep Learning-Based Prediction of the T Cell Receptor–Antigen Binding Specificity. *Nature Machine Intelligence* (2021) 3(10):864-75. doi: 10.1038/s42256-021-00383-2.

12. Kim S, Kim HS, Kim E, Lee MG, Shin EC, Paik S, et al. Neopepsee: Accurate Genome-Level Prediction of Neoantigens by Harnessing Sequence and Amino Acid Immunogenicity Information. *Annals of oncology : official journal of the European Society for Medical Oncology* (2018) 29(4):1030-6. Epub 2018/01/24. doi: 10.1093/annonc/mdy022.

13. Smith CC, Chai S, Washington AR, Lee SJ, Landoni E, Field K, et al. Machine-Learning Prediction of Tumor Antigen Immunogenicity in the Selection of Therapeutic Epitopes. *Cancer immunology research* (2019) 7(10):1591-604. Epub 2019/09/14. doi: 10.1158/2326-6066.cir-19-0155.

14. Zhou C, Wei Z, Zhang Z, Zhang B, Zhu C, Chen K, et al. Ptuneos: Prioritizing Tumor Neoantigens from Next-Generation Sequencing Data. *Genome Med* (2019) 11(1):67. Epub 2019/11/02. doi: 10.1186/s13073-019-0679-x.

15. Carreno BM, Magrini V, Becker-Hapak M, Kaabinejadian S, Hundal J, Petti AA, et al. Cancer Immunotherapy. A Dendritic Cell Vaccine Increases the Breadth and Diversity of Melanoma Neoantigen-Specific T Cells. *Science (New York, NY)* (2015) 348(6236):803-8. Epub 2015/04/04. doi: 10.1126/science.aaa3828.

16. Wang G, Wan H, Jian X, Li Y, Ouyang J, Tan X, et al. Ineo-Epp: A Novel T-Cell Hla Class-I Immunogenicity or Neoantigenic Epitope Prediction Method Based on Sequence-Related Amino Acid Features. *BioMed research international* (2020) 2020:5798356. Epub 2020/07/07. doi: 10.1155/2020/5798356.

17. Bjerregaard AM, Nielsen M, Jurtz V, Barra CM, Hadrup SR, Szallasi Z, et al. An Analysis of Natural T Cell Responses to Predicted Tumor Neoepitopes. *Frontiers in immunology* (2017) 8:1566. Epub 2017/12/01. doi: 10.3389/fimmu.2017.01566.

18. Gartner JJ, Parkhurst MR, Gros A, Tran E, Jafferji MS, Copeland A, et al. A Machine Learning Model for Ranking Candidate Hla Class I Neoantigens Based on Known Neoepitopes from Multiple Human Tumor Types. *Nature cancer* (2021) 2(5):563-74. Epub 2021/12/21. doi: 10.1038/s43018-021-00197-6.

19. Koboldt DC, Zhang Q, Larson DE, Shen D, McLellan MD, Lin L, et al. Varscan 2: Somatic Mutation and Copy Number Alteration Discovery in Cancer by Exome Sequencing. *Genome research* (2012) 22(3):568-76. Epub 2012/02/04. doi: 10.1101/gr.129684.111.

20. Larson DE, Harris CC, Chen K, Koboldt DC, Abbott TE, Dooling DJ, et al. Somaticsniper: Identification of Somatic Point Mutations in Whole Genome Sequencing Data. *Bioinformatics (Oxford, England)* (2012) 28(3):311-7. Epub 2011/12/14. doi: 10.1093/bioinformatics/btr665.

21. Xu F, Wang W, Wang P, Jun Li M, Chung Sham P, Wang J. A Fast and Accurate Snp Detection Algorithm for Next-Generation Sequencing Data. *Nat Commun* (2012) 3:1258. Epub 2012/12/06. doi: 10.1038/ncomms2256.

22. Cibulskis K, Lawrence MS, Carter SL, Sivachenko A, Jaffe D, Sougnez C, et al. Sensitive Detection of Somatic Point Mutations in Impure and Heterogeneous Cancer Samples. *Nat Biotechnol* (2013) 31(3):213-9. Epub 2013/02/12. doi: 10.1038/nbt.2514.

23. Hansen NF, Gartner JJ, Mei L, Samuels Y, Mullikin JC. Shimmer: Detection of Genetic Alterations in Tumors Using Next-Generation Sequence Data. *Bioinformatics (Oxford, England)* (2013) 29(12):1498-503. Epub 2013/04/27. doi: 10.1093/bioinformatics/btt183.

24. Shiraishi Y, Sato Y, Chiba K, Okuno Y, Nagata Y, Yoshida K, et al. An Empirical Bayesian Framework for Somatic Mutation Detection from Cancer Genome Sequencing Data. *Nucleic acids research* (2013) 41(7):e89. Epub 2013/03/09. doi: 10.1093/nar/gkt126.

25. Radenbaugh AJ, Ma S, Ewing A, Stuart JM, Collisson EA, Zhu J, et al. Radia: Rna and DNA Integrated Analysis for Somatic Mutation Detection. *PLoS One* (2014) 9(11):e111516. Epub 2014/11/19. doi: 10.1371/journal.pone.0111516.

26. Lai Z, Markovets A, Ahdesmaki M, Chapman B, Hofmann O, McEwen R, et al. Vardict: A Novel and Versatile Variant Caller for Next-Generation Sequencing in Cancer Research. *Nucleic acids research* (2016) 44(11):e108-e. Epub 04/07. doi: 10.1093/nar/gkw227.

27. Narzisi G, Corvelo A, Arora K, Bergmann EA, Shah M, Musunuri R, et al. Genome-Wide Somatic Variant Calling Using Localized Colored De Bruijn Graphs. *Communications biology* (2018) 1:20. Epub 2018/10/03. doi: 10.1038/s42003-018-0023-9.

28. Kim S, Scheffler K, Halpern AL, Bekritsky MA, Noh E, Källberg M, et al. Strelka2: Fast and Accurate Calling of Germline and Somatic Variants. *Nature methods* (2018) 15(8):591-4. Epub 2018/07/18. doi: 10.1038/s41592-018-0051-x.

29. Mose LE, Perou CM, Parker JS. Improved Indel Detection in DNA and Rna Via Realignment with Abra2. *Bioinformatics (Oxford, England)* (2019) 35(17):2966-73. Epub 2019/01/17. doi: 10.1093/bioinformatics/btz033.

30. Ye K, Schulz MH, Long Q, Apweiler R, Ning Z. Pindel: A Pattern Growth Approach to Detect Break Points of Large Deletions and Medium Sized Insertions from Paired-End Short Reads. *Bioinformatics (Oxford, England)* (2009) 25(21):2865-71. Epub 2009/06/30. doi: 10.1093/bioinformatics/btp394.

31. Layer RM, Chiang C, Quinlan AR, Hall IM. Lumpy: A Probabilistic Framework for Structural Variant Discovery. *Genome biology* (2014) 15(6):R84-R. doi: 10.1186/gb-2014-15-6-r84.

32. Chen X, Schulz-Trieglaff O, Shaw R, Barnes B, Schlesinger F, Källberg M, et al. Manta: Rapid Detection of Structural Variants and Indels for Germline and Cancer Sequencing Applications. *Bioinformatics (Oxford, England)* (2016) 32(8):1220-2. Epub 2015/12/10. doi: 10.1093/bioinformatics/btv710.

33. Wang X, Luan Y, Yue F. Eaglec: A Deep-Learning Framework for Detecting a Full Range of Structural Variations from Bulk and Single-Cell Contact Maps. *Science advances* (2022) 8(24):eabn9215. Epub 2022/06/16. doi: 10.1126/sciadv.abn9215.

34. McPherson A, Hormozdiari F, Zayed A, Giuliany R, Ha G, Sun MG, et al. Defuse: An Algorithm for Gene Fusion Discovery in Tumor Rna-Seq Data. *PLoS computational biology* (2011) 7(5):e1001138. Epub 2011/06/01. doi: 10.1371/journal.pcbi.1001138.

35. Jia W, Qiu K, He M, Song P, Zhou Q, Zhou F, et al. Soapfuse: An Algorithm for Identifying Fusion Transcripts from Paired-End Rna-Seq Data. *Genome biology* (2013) 14(2):R12. Epub 2013/02/16. doi: 10.1186/gb-2013-14-2-r12.

36. Davidson NM, Majewski IJ, Oshlack A. Jaffa: High Sensitivity Transcriptome-Focused Fusion Gene Detection. *Genome Med* (2015) 7(1):43. Epub 2015/05/29. doi: 10.1186/s13073-015-0167-x.

37. Zhao J, Chen Q, Wu J, Han P, Song X. Gfusion: An Effective Algorithm to Identify Fusion Genes from Cancer Rna-Seq Data. *Sci Rep* (2017) 7(1):6880. Epub 2017/08/02. doi: 10.1038/s41598-017-07070-6.

38. Chiu R, Nip KM, Birol I. Fusion-Bloom: Fusion Detection in Assembled Transcriptomes. *Bioinformatics (Oxford, England)* (2020) 36(7):2256-7. Epub 2019/12/04. doi: 10.1093/bioinformatics/btz902.

39. Jayasinghe RG, Cao S, Gao Q, Wendl MC, Vo NS, Reynolds SM, et al. Systematic Analysis of Splice-Site-Creating Mutations in Cancer. *Cell reports* (2018) 23(1):270-81.e3. Epub 2018/04/05. doi: 10.1016/j.celrep.2018.03.052.

40. Cheng J, Nguyen TYD, Cygan KJ, Çelik MH, Fairbrother WG, Avsec Ž, et al. Mmsplice: Modular Modeling Improves the Predictions of Genetic Variant Effects on Splicing. *Genome biology* (2019) 20(1):48. Epub 2019/03/03. doi: 10.1186/s13059-019-1653-z.

41. Li HD, Funk CC, Price ND. Iread: A Tool for Intron Retention Detection from Rna-Seq Data. *BMC genomics* (2020) 21(1):128. Epub 2020/02/08. doi: 10.1186/s12864-020-6541-0.

42. Wang Z, Lian J, Li Q, Zhang P, Zhou Y, Zhan X, et al. Res-Scanner: A Software Package for Genome-Wide Identification of Rna-Editing Sites. *GigaScience* (2016) 5(1):37. Epub 2016/08/20. doi: 10.1186/s13742-016-0143-4.

43. John D, Weirick T, Dimmeler S, Uchida S. Rnaeditor: Easy Detection of Rna Editing Events and the Introduction of Editing Islands. *Briefings in bioinformatics* (2017) 18(6):993-1001. Epub 2016/10/04. doi: 10.1093/bib/bbw087.

44. Ouyang Z, Liu F, Zhao C, Ren C, An G, Mei C, et al. Accurate Identification of Rna Editing Sites from Primitive Sequence with Deep Neural Networks. *Sci Rep* (2018) 8(1):6005. Epub 2018/04/18. doi: 10.1038/s41598-018-24298-y.

45. Boegel S, Löwer M, Schäfer M, Bukur T, de Graaf J, Boisguérin V, et al. Hla Typing from Rna-Seq Sequence Reads. *Genome Med* (2012) 4(12):102. Epub 2012/12/25. doi: 10.1186/gm403.

46. Cao H, Wu J, Wang Y, Jiang H, Zhang T, Liu X, et al. An Integrated Tool to Study Mhc Region: Accurate Snv Detection and Hla Genes Typing in Human Mhc Region Using Targeted High-Throughput Sequencing. *PLoS One* (2013) 8(7):e69388. Epub 2013/07/31. doi: 10.1371/journal.pone.0069388.

47. Kim HJ, Pourmand N. Hla Typing from Rna-Seq Data Using Hierarchical Read Weighting [Corrected]. *PLoS One* (2013) 8(6):e67885. Epub 2013/07/11. doi: 10.1371/journal.pone.0067885.

48. Szolek A, Schubert B, Mohr C, Sturm M, Feldhahn M, Kohlbacher O. Optitype: Precision Hla Typing from Next-Generation Sequencing Data. *Bioinformatics (Oxford, England)* (2014) 30(23):3310-6. Epub 2014/08/22. doi: 10.1093/bioinformatics/btu548.

49. Liu C, Yang X, Duffy B, Mohanakumar T, Mitra RD, Zody MC, et al. Athlates: Accurate Typing of Human Leukocyte Antigen through Exome Sequencing. *Nucleic acids research* (2013) 41(14):e142. Epub 2013/06/12. doi: 10.1093/nar/gkt481.

50. Shukla SA, Rooney MS, Rajasagi M, Tiao G, Dixon PM, Lawrence MS, et al. Comprehensive Analysis of Cancer-Associated Somatic Mutations in Class I Hla Genes. *Nat Biotechnol* (2015) 33(11):1152-8. Epub 2015/09/16. doi: 10.1038/nbt.3344.

51. Warren RL, Choe G, Freeman DJ, Castellarin M, Munro S, Moore R, et al. Derivation of Hla Types from Shotgun Sequence Datasets. *Genome Med* (2012) 4(12):95. Epub 2012/12/12. doi: 10.1186/gm396.

52. Huang Y, Yang J, Ying D, Zhang Y, Shotelersuk V, Hirankarn N, et al. Hlareporter: A Tool for Hla Typing from Next Generation Sequencing Data. *Genome Med* (2015) 7(1):25. Epub 2015/04/25. doi: 10.1186/s13073-015-0145-3.

53. Nariai N, Kojima K, Saito S, Mimori T, Sato Y, Kawai Y, et al. Hla-Vbseq: Accurate Hla Typing at Full Resolution from Whole-Genome Sequencing Data. *BMC genomics* (2015) 16 Suppl 2(Suppl 2):S7. Epub 2015/02/25. doi: 10.1186/1471-2164-16-s2-s7.

54. Ka S, Lee S, Hong J, Cho Y, Sung J, Kim HN, et al. Hlascan: Genotyping of the Hla Region Using Next-Generation Sequencing Data. *BMC bioinformatics* (2017) 18(1):258. Epub 2017/05/14. doi: 10.1186/s12859-017-1671-3.

55. Buchkovich ML, Brown CC, Robasky K, Chai S, Westfall S, Vincent BG, et al. Hlaprofiler Utilizes K-Mer Profiles to Improve Hla Calling Accuracy for Rare and Common Alleles in Rna-Seq Data. *Genome Med* (2017) 9(1):86. Epub 2017/09/29. doi: 10.1186/s13073-017-0473-6.

56. Xie C, Yeo ZX, Wong M, Piper J, Long T, Kirkness EF, et al. Fast and Accurate Hla Typing from Short-Read Next-Generation Sequence Data with Xhla. *Proc Natl Acad Sci U S A* (2017) 114(30):8059-64. Epub 2017/07/05. doi: 10.1073/pnas.1707945114.

57. Kawaguchi S, Higasa K, Shimizu M, Yamada R, Matsuda F. Hla-Hd: An Accurate Hla Typing Algorithm for Next-Generation Sequencing Data. *Human mutation* (2017) 38(7):788-97. Epub 2017/04/19. doi: 10.1002/humu.23230.

58. Lee H, Kingsford C. Kourami: Graph-Guided Assembly for Novel Human Leukocyte Antigen Allele Discovery. *Genome biology* (2018) 19(1):16. Epub 2018/02/09. doi: 10.1186/s13059-018-1388-2.

59. Bai Y, Wang D, Fury W. Phlat: Inference of High-Resolution Hla Types from Rna and Whole Exome Sequencing. *Hla Typing*. Springer (2018). p. 193-201.

60. Bassani-Sternberg M. Mass Spectrometry Based Immunopeptidomics for the Discovery of Cancer Neoantigens. *Methods in molecular biology (Clifton, NJ)* (2018) 1719:209-21. Epub 2018/02/25. doi: 10.1007/978-1-4939-7537-2_14.

61. Orenbuch R, Filip I, Comito D, Shaman J, Pe'er I, Rabadan R. Arcashla: High-Resolution Hla Typing from Rnaseq. *Bioinformatics (Oxford, England)* (2020) 36(1):33-40. Epub 2019/06/08. doi: 10.1093/bioinformatics/btz474.

62. Nielsen M, Lundegaard C, Lund O. Prediction of Mhc Class Ii Binding Affinity Using Smm-Align, a Novel Stabilization Matrix Alignment Method. *BMC bioinformatics* (2007) 8:238. Epub 2007/07/05. doi: 10.1186/1471-2105-8-238.

63. Sturniolo T, Bono E, Ding J, Raddrizzani L, Tuereci O, Sahin U, et al. Generation of Tissue-Specific and Promiscuous Hla Ligand Databases Using DNA Microarrays and Virtual Hla Class Ii Matrices. *Nat Biotechnol* (1999) 17(6):555-61. Epub 1999/06/29. doi: 10.1038/9858.

64. Kim Y, Sidney J, Pinilla C, Sette A, Peters B. Derivation of an Amino Acid Similarity Matrix for Peptide: Mhc Binding and Its Application as a Bayesian Prior. *BMC bioinformatics* (2009) 10:394. Epub 2009/12/02. doi: 10.1186/1471-2105-10-394.

65. Singh H, Raghava GP. Propred: Prediction of Hla-Dr Binding Sites. *Bioinformatics (Oxford, England)* (2001) 17(12):1236-7. Epub 2001/12/26. doi: 10.1093/bioinformatics/17.12.1236.

66. Zhang H, Lund O, Nielsen M. The Pickpocket Method for Predicting Binding Specificities for Receptors Based on Receptor Pocket Similarities: Application to Mhc-Peptide Binding. *Bioinformatics (Oxford, England)* (2009) 25(10):1293-9. Epub 2009/03/20. doi: 10.1093/bioinformatics/btp137.

67. Reche PA, Glutting JP, Reinherz EL. Prediction of Mhc Class I Binding Peptides Using Profile Motifs. *Human immunology* (2002) 63(9):701-9. Epub 2002/08/15. doi: 10.1016/s0198-8859(02)00432-9.

68. Karosiene E, Lundegaard C, Lund O, Nielsen M. Netmhccons: A Consensus Method for the Major Histocompatibility Complex Class I Predictions. *Immunogenetics* (2012) 64(3):177-86. Epub 2011/10/20. doi: 10.1007/s00251-011-0579-8.

69. Wan J, Liu W, Xu Q, Ren Y, Flower DR, Li T. Svrmhc Prediction Server for Mhc-Binding Peptides. *BMC bioinformatics* (2006) 7:463. Epub 2006/10/25. doi: 10.1186/1471-2105-7-463.

70. Andreatta M, Nielsen M. Gapped Sequence Alignment Using Artificial Neural Networks: Application to the Mhc Class I System. *Bioinformatics (Oxford, England)* (2016) 32(4):511-7. Epub 2015/10/31. doi: 10.1093/bioinformatics/btv639.

71. Sidney J, Assarsson E, Moore C, Ngo S, Pinilla C, Sette A, et al. Quantitative Peptide Binding Motifs for 19 Human and Mouse Mhc Class I Molecules Derived Using Positional Scanning Combinatorial Peptide Libraries. *Immunome research* (2008) 4:2. Epub 2008/01/29. doi: 10.1186/1745-7580-4-2.

72. Bhattacharya R, Tokheim C, Sivakumar A, Guthrie VB, Anagnostou V, Velculescu VE, et al. Prediction of Peptide Binding to Mhc Class I Proteins in the Age of Deep Learning. *bioRxiv : the preprint server for biology* (2017):154757. doi: 10.1101/154757.

73. Andreatta M, Schafer-Nielsen C, Lund O, Buus S, Nielsen M. Nnalign: A Web-Based Prediction Method Allowing Non-Expert End-User Discovery of Sequence Motifs in Quantitative Peptide Data. *PLoS One* (2011) 6(11):e26781. Epub 2011/11/11. doi: 10.1371/journal.pone.0026781.

74. Bassani-Sternberg M, Chong C, Guillaume P, Solleder M, Pak H, Gannon PO, et al. Deciphering Hla-I Motifs across Hla Peptidomes Improves Neo-Antigen Predictions and Identifies Allostery Regulating Hla Specificity. *PLoS computational biology* (2017) 13(8):e1005725. Epub 2017/08/24. doi: 10.1371/journal.pcbi.1005725.

75. Bordner AJ, Mittelmann HD. Multirta: A Simple yet Reliable Method for Predicting Peptide Binding Affinities for Multiple Class Ii Mhc Allotypes. *BMC bioinformatics* (2010) 11:482. Epub 2010/09/28. doi: 10.1186/1471-2105-11-482.

76. Han Y, Kim D. Deep Convolutional Neural Networks for Pan-Specific Peptide-Mhc Class I Binding Prediction. *BMC bioinformatics* (2017) 18(1):585. Epub 2017/12/29. doi: 10.1186/s12859-017-1997-x.

77. Zhang L, Chen Y, Wong HS, Zhou S, Mamitsuka H, Zhu S. Tepitopepan: Extending Tepitope for Peptide Binding Prediction Covering over 700 Hla-Dr Molecules. *PLoS One* (2012) 7(2):e30483. Epub 2012/03/03. doi: 10.1371/journal.pone.0030483.

78. O'Donnell TJ, Rubinsteyn A, Bonsack M, Riemer AB, Laserson U, Hammerbacher J. Mhcflurry: Open-Source Class I Mhc Binding Affinity Prediction. *Cell systems* (2018) 7(1):129-32.e4. Epub 2018/07/02. doi: 10.1016/j.cels.2018.05.014.

79. Shen WJ, Zhang S, Wong HS. An Effective and Effecient Peptide Binding Prediction Approach for a Broad Set of Hla-Dr Molecules Based on Ordered Weighted Averaging of Binding Pocket Profiles. *Proteome science* (2013) 11(Suppl 1):S15. Epub 2014/02/26. doi: 10.1186/1477-5956-11-s1-s15.

80. Phloyphisut P, Pornputtanapong N, Sriswasdi S, Chuangsuwanich E. Mhcseqnet: A Deep Neural Network Model for Universal Mhc Binding Prediction. *bioRxiv : the preprint server for biology* (2018):371591. doi: 10.1101/371591.

81. Jensen KK, Andreatta M, Marcatili P, Buus S, Greenbaum JA, Yan Z, et al. Improved Methods for Predicting Peptide Binding Affinity to Mhc Class Ii Molecules. *Immunology* (2018) 154(3):394-406. Epub 2018/01/10. doi: 10.1111/imm.12889.

82. Degoot AM, Chirove F, Ndifon W. Trans-Allelic Model for Prediction of Peptide:Mhc-Ii Interactions. *Frontiers in immunology* (2018) 9.

83. Racle J, Michaux J, Rockinger GA, Arnaud M, Bobisse S, Chong C, et al. Deep Motif Deconvolution of Hla-Ii Peptidomes for Robust Class Ii Epitope Predictions. *bioRxiv : the preprint server for biology* (2019):539338. doi: 10.1101/539338.

84. Mei S, Li F, Xiang D, Ayala R, Faridi P, Webb GI, et al. Anthem: A User Customised Tool for Fast and Accurate Prediction of Binding between Peptides and Hla Class I Molecules. *Briefings in bioinformatics* (2021) 22(5). Epub 2021/01/18. doi: 10.1093/bib/bbaa415.

85. Chen B, Khodadoust MS, Olsson N, Wagar LE, Fast E, Liu CL, et al. Predicting Hla Class Ii Antigen Presentation through Integrated Deep Learning. *Nat Biotechnol* (2019) 37(11):1332-43. Epub 2019/10/16. doi: 10.1038/s41587-019-0280-2.

86. Bravi B, Tubiana J, Cocco S, Monasson R, Mora T, Walczak AM. Rbm-Mhc: A Semi-Supervised Machine-Learning Method for Sample-Specific Prediction of Antigen Presentation by Hla-I Alleles. *Cell systems* (2021) 12(2):195-202.e9. Epub 2020/12/19. doi: 10.1016/j.cels.2020.11.005.

87. Abelin JG, Harjanto D, Malloy M, Suri P, Colson T, Goulding SP, et al. Defining Hla-Ii Ligand Processing and Binding Rules with Mass Spectrometry Enhances Cancer Epitope Prediction. *Immunity* (2019) 51(4):766-79.e17. Epub 2019/09/10. doi: 10.1016/j.immuni.2019.08.012.

88. Hao Q, Wei P, Shu Y, Zhang YG, Xu H, Zhao JN. Improvement of Neoantigen Identification through Convolution Neural Network. *Frontiers in immunology* (2021) 12:682103. Epub 2021/06/12. doi: 10.3389/fimmu.2021.682103.

89. Yang X, Zhao L, Wei F, Li J. Deepnetbim: Deep Learning Model for Predicting Hla-Epitope Interactions Based on Network Analysis by Harnessing Binding and Immunogenicity Information. *BMC bioinformatics* (2021) 22(1):231. Epub 2021/05/07. doi: 10.1186/s12859-021-04155-y.

90. Liu Z, Jin J, Cui Y, Xiong Z, Nasiri A, Zhao Y, et al. Deepseqpanii: An Interpretable Recurrent Neural Network Model with Attention Mechanism for Peptide-Hla Class Ii Binding Prediction. *IEEE/ACM transactions on computational biology and bioinformatics* (2022) 19(4):2188-96. Epub 2021/04/23. doi: 10.1109/tcbb.2021.3074927.

91. Wang F, Wang H, Wang L, Lu H, Qiu S, Zang T, et al. Mhcroberta: Pan-Specific Peptide-Mhc Class I Binding Prediction through Transfer Learning with Label-Agnostic Protein Sequences. *Briefings in bioinformatics* (2022) 23(3). Epub 2022/04/21. doi: 10.1093/bib/bbab595.
